# Supplementary material for: Capturing site-specific heterogeneity with large-scale N-glycoproteome analysis
Source: Nat Commun. 2019 Mar 21;10:1311. doi: 10.1038/s41467-019-09222-w (PMC6428843; doi:10.1038/s41467-019-09222-w)
Supplement: Supplementary file 1 — Supplementary Information [file 41467_2019_9222_MOESM1_ESM.docx]

**SUPPLEMENTARY INFORMATION**

**Capturing site-specific heterogeneity with large-scale N-glycoproteome analysis**

Riley et al.

**Supplementary Tables and Figures**

**Supplementary Table 1.** Glycan identities for node labels from Supplementary Figure 11.

**Supplementary Table 2.** Glycan identities for the co-occurrence heat map from Figure 2g Supplementary Figure 12.

**Supplementary Table 3.** Glycan identities for node labels from Figure 3 and Supplementary Figure 13.

**Supplementary Figure 1.** Comparing ETD, ETD with supplemental activation using higher energy collisional dissociation (EThcD) and AI-ETD.

**Supplementary Figure 2.** Distributions of fragment ions generated by AI-ETD (green) and HCD (dark blue).

**Supplementary Figure 3.** Percent of spectra containing specific oxonium and Y-type ions.

**Supplementary Figure 4.** Using oxonium ions to differentiate glycan isomers.

**Supplementary Figure 5.** HCD performance characteristics.

**Supplementary Figure 6.** Mass and m/z distributions of glycoproteomics data.

**Supplementary Figure 7.** Workflow for in-depth profiling of the glycoproteome via intact glycopeptides with AI-ETD.

**Supplementary Figure 8.** Comparison of glycosites to large-scale deglycoproteomic experiments.

**Supplementary Figure 9.** N-glycosites characterized in integrin alpha-1 (GN: Itga1, UniProt: Q3V3R4) in mouse brain.

**Supplementary Figure 10.** Gene ontology functional categories enriched terms for identified glycoproteins.

**Supplementary Figure 11.** A larger version of the glycoprotein-glycan map in Figure 2f.

**Supplementary Figure 12.** A larger version of the co-occurrence heat map in Figure 2g.

**Supplementary Figure 13.** Glycan co-occurrence networks for a specific glycan and a class of glycans.

**Supplementary Figure 14.** Arc plot representing glycan microheterogeneity for glycosites with two glycans per site.

**Supplementary Figure 15.** Arc plot representing glycan microheterogeneity for glycosites with three glycans per site.

**Supplementary Figure 16.** Arc plot representing glycan microheterogeneity for glycosites with four glycans per site.

**Supplementary Figure 17.** Arc plot representing glycan microheterogeneity for glycosites with five glycans per site.

**Supplementary Figure 18.** Mass differences between glycans that occupy the same glycosite.

**Supplementary Figure 19.** Protein sidekick-2: relatively high number of glycosites with relatively low glycan microheterogeneity.

**Supplementary Figure 20.** Secreted protein acidic and rich in cysteine (SPARC): only one glycosite with moderate glycan microheterogeneity.

**Supplementary Figure 21.** Sodium/potassium transporting-ATPase β2 subunit (Atp1b2): glycosites with varying degrees of glycan microheterogeneity.

**Supplementary Figure 22.** Heat map of the number of subcellular groups (derived from GO cellular component terms) for identified glycoproteins.

**Supplementary Table 1.** Glycan identities for node labels from Supplementary Figure 11.

| **Node** | **Glycan** |
| --- | --- |
| 1 | HexNAc(1) |
| 2 | HexNAc(2) |
| 3 | HexNAc(2)Fuc(1) |
| 4 | HexNAc(2)Hex(1) |
| 5 | HexNAc(2)Hex(1)Fuc(1) |
| 6 | HexNAc(2)Hex(2) |
| 7 | HexNAc(2)Hex(2)Fuc(1) |
| 8 | HexNAc(2)Hex(3) |
| 9 | HexNAc(2)Hex(3)Fuc(1) |
| 10 | HexNAc(3)Hex(4)Fuc(1)NeuAc(1) |
| 11 | HexNAc(3)Hex(4)Fuc(2)NeuAc(1) |
| 12 | HexNAc(3)Hex(4)NeuAc(1) |
| 13 | HexNAc(3)Hex(5)Fuc(1)NeuAc(1) |
| 14 | HexNAc(3)Hex(5)NeuAc(1) |
| 15 | HexNAc(3)Hex(6)Fuc(1)NeuAc(1) |
| 16 | HexNAc(3)Hex(6)NeuAc(1) |
| 17 | HexNAc(4)Hex(3)NeuAc(1) |
| 18 | HexNAc(4)Hex(4)Fuc(1)NeuAc(1) |
| 19 | HexNAc(4)Hex(4)NeuAc(1) |
| 20 | HexNAc(4)Hex(5)Fuc(1)NeuAc(1) |
| 21 | HexNAc(4)Hex(5)Fuc(1)NeuAc(2) |
| 22 | HexNAc(4)Hex(5)Fuc(2)NeuAc(1) |
| 23 | HexNAc(4)Hex(5)Fuc(3)NeuAc(1) |
| 24 | HexNAc(4)Hex(5)NeuAc(1) |
| 25 | HexNAc(4)Hex(6)Fuc(1)NeuAc(1) |
| 26 | HexNAc(4)Hex(6)NeuAc(1) |
| 27 | HexNAc(4)Hex(7)NeuAc(1) |
| 28 | HexNAc(5)Hex(3)Fuc(1)NeuAc(1) |
| 29 | HexNAc(5)Hex(4)Fuc(1)NeuAc(1) |
| 30 | HexNAc(5)Hex(4)NeuAc(1) |
| 31 | HexNAc(5)Hex(4)NeuAc(2) |
| 32 | HexNAc(5)Hex(5)Fuc(1)NeuAc(2) |
| 33 | HexNAc(5)Hex(5)Fuc(2)NeuAc(1) |
| 34 | HexNAc(5)Hex(6)NeuAc(2) |
| 35 | HexNAc(6)Hex(3)Fuc(1)NeuAc(1) |
| 36 | HexNAc(6)Hex(3)Fuc(1)NeuAc(2) |
| 37 | HexNAc(6)Hex(5)Fuc(1)NeuAc(2) |
| 38 | HexNAc(6)Hex(5)Fuc(2)NeuAc(1) |
| **Node** | **Glycan** |
| 39 | HexNAc(6)Hex(6)Fuc(2)NeuAc(1) |
| 40 | HexNAc(6)Hex(6)NeuAc(1) |
| 41 | HexNAc(6)Hex(7)Fuc(3)NeuAc(1) |
| 42 | HexNAc(6)Hex(7)NeuAc(3) |
| 43 | HexNAc(6)Hex(7)NeuAc(4) |
| 44 | HexNAc(6)Hex(9)Fuc(1)NeuAc(2) |
| 45 | HexNAc(7)Hex(8)Fuc(1)NeuAc(1) |
| 46 | HexNAc(3)Hex(3) |
| 47 | HexNAc(3)Hex(4) |
| 48 | HexNAc(3)Hex(5) |
| 49 | HexNAc(3)Hex(6) |
| 50 | HexNAc(4)Hex(3) |
| 51 | HexNAc(4)Hex(4) |
| 52 | HexNAc(4)Hex(5) |
| 53 | HexNAc(4)Hex(6) |
| 54 | HexNAc(4)Hex(7) |
| 55 | HexNAc(5)Hex(3) |
| 56 | HexNAc(5)Hex(4) |
| 57 | HexNAc(5)Hex(6) |
| 58 | HexNAc(5)Hex(8) |
| 59 | HexNAc(6)Hex(3) |
| 60 | HexNAc(6)Hex(4) |
| 61 | HexNAc(6)Hex(5) |
| 62 | HexNAc(6)Hex(7) |
| 63 | HexNAc(6)Hex(9) |
| 64 | HexNAc(7)Hex(3) |
| 65 | HexNAc(7)Hex(4) |
| 66 | HexNAc(7)Hex(6) |
| 67 | HexNAc(8)Hex(3) |
| 68 | HexNAc(8)Hex(4) |
| 69 | HexNAc(8)Hex(5) |
| 70 | HexNAc(8)Hex(8) |
| 71 | HexNAc(9)Hex(10) |
| 72 | HexNAc(9)Hex(3) |
| 73 | HexNAc(9)Hex(6) |
| 74 | HexNAc(2)Hex(4)Fuc(1) |
| 75 | HexNAc(2)Hex(5)Fuc(1) |
| 76 | HexNAc(2)Hex(6)Fuc(1) |
| **Node** | **Glycan** |
| 77 | HexNAc(3)Hex(3)Fuc(1) |
| 78 | HexNAc(3)Hex(4)Fuc(1) |
| 79 | HexNAc(3)Hex(4)Fuc(2) |
| 80 | HexNAc(3)Hex(5)Fuc(1) |
| 81 | HexNAc(3)Hex(6)Fuc(1) |
| 82 | HexNAc(4)Hex(3)Fuc(1) |
| 83 | HexNAc(4)Hex(4)Fuc(1) |
| 84 | HexNAc(4)Hex(4)Fuc(2) |
| 85 | HexNAc(4)Hex(5)Fuc(1) |
| 86 | HexNAc(4)Hex(5)Fuc(2) |
| 87 | HexNAc(4)Hex(6)Fuc(1) |
| 88 | HexNAc(4)Hex(6)Fuc(2) |
| 89 | HexNAc(4)Hex(7)Fuc(1) |
| 90 | HexNAc(5)Hex(3)Fuc(1) |
| 91 | HexNAc(5)Hex(3)Fuc(2) |
| 92 | HexNAc(5)Hex(4)Fuc(1) |
| 93 | HexNAc(5)Hex(4)Fuc(2) |
| 94 | HexNAc(5)Hex(5)Fuc(1) |
| 95 | HexNAc(5)Hex(6)Fuc(4) |
| 96 | HexNAc(5)Hex(8)Fuc(1) |
| 97 | HexNAc(6)Hex(4)Fuc(1) |
| **Node** | **Glycan** |
| 98 | HexNAc(6)Hex(4)Fuc(2) |
| 99 | HexNAc(6)Hex(6)Fuc(2) |
| 100 | HexNAc(6)Hex(7)Fuc(3) |
| 101 | HexNAc(7)Hex(3)Fuc(1) |
| 102 | HexNAc(7)Hex(7)Fuc(1) |
| 103 | HexNAc(8)Hex(5)Fuc(1) |
| 104 | HexNAc(8)Hex(9)Fuc(1) |
| 105 | HexNAc(9)Hex(3)Fuc(1) |
| 106 | HexNAc(9)Hex(6)Fuc(1) |
| 107 | HexNAc(9)Hex(9)Fuc(1) |
| 108 | HexNAc(2)Hex(12) |
| 109 | HexNAc(2)Hex(11) |
| 110 | HexNAc(2)Hex(10) |
| 111 | HexNAc(2)Hex(9) |
| 112 | HexNAc(2)Hex(8) |
| 113 | HexNAc(2)Hex(7) |
| 114 | HexNAc(2)Hex(6) |
| 115 | HexNAc(2)Hex(5) |
| 116 | HexNAc(2)Hex(4) |
| 117 | HexNAc(2)Hex(6)Phospho(1) |

**Supplementary Table 2.** Glycan identities for the co-occurrence heat map from **Figure 2g** **Supplementary Figure 12**.

| **Order#** | **Glycan** |
| --- | --- |
| 1 | HexNAc(1) |
| 2 | HexNAc(2) |
| 3 | HexNAc(2)Fuc(1) |
| 4 | HexNAc(2)Hex(1) |
| 5 | HexNAc(2)Hex(1)Fuc(1) |
| 6 | HexNAc(2)Hex(2) |
| 7 | HexNAc(2)Hex(2)Fuc(1) |
| 8 | HexNAc(2)Hex(3) |
| 9 | HexNAc(2)Hex(3)Fuc(1) |
| 10 | HexNAc(2)Hex(4) |
| 11 | HexNAc(2)Hex(5) |
| 12 | HexNAc(2)Hex(6) |
| 13 | HexNAc(2)Hex(7) |
| 14 | HexNAc(2)Hex(8) |
| 15 | HexNAc(2)Hex(9) |
| 16 | HexNAc(2)Hex(10) |
| 17 | HexNAc(2)Hex(11) |
| 18 | HexNAc(2)Hex(12) |
| 19 | HexNAc(2)Hex(6)Phospho(1) |
| 20 | HexNAc(3)Hex(3) |
| 21 | HexNAc(3)Hex(4) |
| 22 | HexNAc(3)Hex(5) |
| 23 | HexNAc(3)Hex(6) |
| 24 | HexNAc(4)Hex(3) |
| 25 | HexNAc(4)Hex(4) |
| 26 | HexNAc(4)Hex(5) |
| 27 | HexNAc(4)Hex(6) |
| 28 | HexNAc(4)Hex(7) |
| 29 | HexNAc(5)Hex(3) |
| 30 | HexNAc(5)Hex(6) |
| 31 | HexNAc(5)Hex(8) |
| 32 | HexNAc(6)Hex(3) |
| 33 | HexNAc(6)Hex(4) |
| 34 | HexNAc(6)Hex(5) |
| 35 | HexNAc(6)Hex(7) |
| 36 | HexNAc(6)Hex(9) |
| 37 | HexNAc(7)Hex(3) |
| 38 | HexNAc(7)Hex(4) |
| **Order#** | **Glycan** |
| 39 | HexNAc(7)Hex(6) |
| 40 | HexNAc(8)Hex(3) |
| 41 | HexNAc(8)Hex(4) |
| 42 | HexNAc(8)Hex(5) |
| 43 | HexNAc(8)Hex(8) |
| 44 | HexNAc(9)Hex(10) |
| 45 | HexNAc(9)Hex(3) |
| 46 | HexNAc(9)Hex(6) |
| 47 | HexNAc(2)Hex(4)Fuc(1) |
| 48 | HexNAc(2)Hex(5)Fuc(1) |
| 49 | HexNAc(2)Hex(6)Fuc(1) |
| 50 | HexNAc(3)Hex(3)Fuc(1) |
| 51 | HexNAc(3)Hex(4)Fuc(1) |
| 52 | HexNAc(3)Hex(4)Fuc(2) |
| 53 | HexNAc(3)Hex(5)Fuc(1) |
| 54 | HexNAc(3)Hex(6)Fuc(1) |
| 55 | HexNAc(4)Hex(3)Fuc(1) |
| 56 | HexNAc(4)Hex(4)Fuc(1) |
| 57 | HexNAc(4)Hex(4)Fuc(2) |
| 58 | HexNAc(4)Hex(5)Fuc(1) |
| 59 | HexNAc(4)Hex(5)Fuc(2) |
| 60 | HexNAc(4)Hex(6)Fuc(1) |
| 61 | HexNAc(4)Hex(6)Fuc(2) |
| 62 | HexNAc(4)Hex(7)Fuc(1) |
| 63 | HexNAc(5)Hex(3)Fuc(1) |
| 64 | HexNAc(5)Hex(3)Fuc(2) |
| 65 | HexNAc(5)Hex(4)Fuc(1) |
| 66 | HexNAc(5)Hex(4)Fuc(2) |
| 67 | HexNAc(5)Hex(5)Fuc(1) |
| 68 | HexNAc(5)Hex(6)Fuc(4) |
| 69 | HexNAc(5)Hex(8)Fuc(1) |
| 70 | HexNAc(6)Hex(4)Fuc(1) |
| 71 | HexNAc(6)Hex(4)Fuc(2) |
| 72 | HexNAc(6)Hex(6)Fuc(2) |
| 73 | HexNAc(6)Hex(7)Fuc(3) |
| 74 | HexNAc(7)Hex(3)Fuc(1) |
| 75 | HexNAc(7)Hex(7)Fuc(1) |
| 76 | HexNAc(8)Hex(9)Fuc(1) |
| **Order#** | **Glycan** |
| 77 | HexNAc(9)Hex(3)Fuc(1) |
| 78 | HexNAc(9)Hex(6)Fuc(1) |
| 79 | HexNAc(9)Hex(9)Fuc(1) |
| 80 | HexNAc(3)Hex(4)Fuc(1)NeuAc(1) |
| 81 | HexNAc(3)Hex(4)Fuc(2)NeuAc(1) |
| 82 | HexNAc(3)Hex(4)NeuAc(1) |
| 83 | HexNAc(3)Hex(5)Fuc(1)NeuAc(1) |
| 84 | HexNAc(3)Hex(5)NeuAc(1) |
| 85 | HexNAc(3)Hex(6)Fuc(1)NeuAc(1) |
| 86 | HexNAc(3)Hex(6)NeuAc(1) |
| 87 | HexNAc(4)Hex(3)NeuAc(1) |
| 88 | HexNAc(4)Hex(4)Fuc(1)NeuAc(1) |
| 89 | HexNAc(4)Hex(4)NeuAc(1) |
| 90 | HexNAc(4)Hex(5)Fuc(1)NeuAc(1) |
| 91 | HexNAc(4)Hex(5)Fuc(1)NeuAc(2) |
| 92 | HexNAc(4)Hex(5)Fuc(2)NeuAc(1) |
| 93 | HexNAc(4)Hex(5)Fuc(3)NeuAc(1) |
| 94 | HexNAc(4)Hex(5)NeuAc(1) |
| 95 | HexNAc(4)Hex(6)Fuc(1)NeuAc(1) |
| 96 | HexNAc(4)Hex(6)NeuAc(1) |
| 97 | HexNAc(4)Hex(7)NeuAc(1) |
| **Order#** | **Glycan** |
| 98 | HexNAc(5)Hex(3)Fuc(1)NeuAc(1) |
| 99 | HexNAc(5)Hex(4)Fuc(1)NeuAc(1) |
| 100 | HexNAc(5)Hex(4)NeuAc(1) |
| 101 | HexNAc(5)Hex(4)NeuAc(2) |
| 102 | HexNAc(5)Hex(5)Fuc(1)NeuAc(2) |
| 103 | HexNAc(5)Hex(5)Fuc(2)NeuAc(1) |
| 104 | HexNAc(5)Hex(6)NeuAc(2) |
| 105 | HexNAc(6)Hex(3)Fuc(1)NeuAc(1) |
| 106 | HexNAc(6)Hex(3)Fuc(1)NeuAc(2) |
| 107 | HexNAc(6)Hex(5)Fuc(1)NeuAc(2) |
| 108 | HexNAc(6)Hex(5)Fuc(2)NeuAc(1) |
| 109 | HexNAc(6)Hex(6)Fuc(2)NeuAc(1) |
| 110 | HexNAc(6)Hex(7)Fuc(3)NeuAc(1) |
| 111 | HexNAc(6)Hex(7)NeuAc(3) |
| 112 | HexNAc(6)Hex(7)NeuAc(4) |
| 113 | HexNAc(6)Hex(9)Fuc(1)NeuAc(2) |
| 114 | HexNAc(7)Hex(8)Fuc(1)NeuAc(1) |

**Supplementary Table 3.** Glycan identities for node labels from **Figure 3** and **Supplementary Figure 13**.

| **Node** | **Glycan** |
| --- | --- |
| 1 | HexNAc(1) |
| 2 | HexNAc(2) |
| 3 | HexNAc(2)Fuc(1) |
| 4 | HexNAc(2)Hex(1) |
| 5 | HexNAc(2)Hex(1)Fuc(1) |
| 6 | HexNAc(2)Hex(2) |
| 7 | HexNAc(2)Hex(2)Fuc(1) |
| 8 | HexNAc(2)Hex(3) |
| 9 | HexNAc(2)Hex(3)Fuc(1) |
| 10 | HexNAc(2)Hex(4) |
| 11 | HexNAc(2)Hex(5) |
| 12 | HexNAc(2)Hex(6) |
| 13 | HexNAc(2)Hex(7) |
| 14 | HexNAc(2)Hex(8) |
| 15 | HexNAc(2)Hex(9) |
| 16 | HexNAc(2)Hex(10) |
| 17 | HexNAc(2)Hex(11) |
| 18 | HexNAc(2)Hex(12) |
| 19 | HexNAc(2)Hex(6)Phospho(1) |
| 20 | HexNAc(3)Hex(3) |
| 21 | HexNAc(3)Hex(4) |
| 22 | HexNAc(3)Hex(5) |
| 23 | HexNAc(3)Hex(6) |
| 24 | HexNAc(4)Hex(3) |
| 25 | HexNAc(4)Hex(4) |
| 26 | HexNAc(4)Hex(5) |
| 27 | HexNAc(4)Hex(6) |
| 28 | HexNAc(4)Hex(7) |
| 29 | HexNAc(5)Hex(3) |
| 30 | HexNAc(5)Hex(4) |
| 31 | HexNAc(5)Hex(6) |
| 32 | HexNAc(5)Hex(8) |
| 33 | HexNAc(6)Hex(3) |
| 34 | HexNAc(6)Hex(4) |
| 35 | HexNAc(6)Hex(5) |
| 36 | HexNAc(6)Hex(7) |
| 37 | HexNAc(6)Hex(9) |
| 38 | HexNAc(7)Hex(3) |
| **Node** | **Glycan** |
| 39 | HexNAc(7)Hex(4) |
| 40 | HexNAc(7)Hex(6) |
| 41 | HexNAc(8)Hex(3) |
| 42 | HexNAc(8)Hex(4) |
| 43 | HexNAc(8)Hex(5) |
| 44 | HexNAc(8)Hex(8) |
| 45 | HexNAc(9)Hex(10) |
| 46 | HexNAc(9)Hex(3) |
| 47 | HexNAc(9)Hex(6) |
| 48 | HexNAc(2)Hex(4)Fuc(1) |
| 49 | HexNAc(2)Hex(5)Fuc(1) |
| 50 | HexNAc(2)Hex(6)Fuc(1) |
| 51 | HexNAc(3)Hex(3)Fuc(1) |
| 52 | HexNAc(3)Hex(4)Fuc(1) |
| 53 | HexNAc(3)Hex(4)Fuc(2) |
| 54 | HexNAc(3)Hex(5)Fuc(1) |
| 55 | HexNAc(3)Hex(6)Fuc(1) |
| 56 | HexNAc(4)Hex(3)Fuc(1) |
| 57 | HexNAc(4)Hex(4)Fuc(1) |
| 58 | HexNAc(4)Hex(4)Fuc(2) |
| 59 | HexNAc(4)Hex(5)Fuc(1) |
| 60 | HexNAc(4)Hex(5)Fuc(2) |
| 61 | HexNAc(4)Hex(6)Fuc(1) |
| 62 | HexNAc(4)Hex(6)Fuc(2) |
| 63 | HexNAc(4)Hex(7)Fuc(1) |
| 64 | HexNAc(5)Hex(3)Fuc(1) |
| 65 | HexNAc(5)Hex(3)Fuc(2) |
| 66 | HexNAc(5)Hex(4)Fuc(1) |
| 67 | HexNAc(5)Hex(4)Fuc(2) |
| 68 | HexNAc(5)Hex(5)Fuc(1) |
| 69 | HexNAc(5)Hex(6)Fuc(4) |
| 70 | HexNAc(5)Hex(8)Fuc(1) |
| 71 | HexNAc(6)Hex(4)Fuc(1) |
| 72 | HexNAc(6)Hex(4)Fuc(2) |
| 73 | HexNAc(6)Hex(6)Fuc(2) |
| 74 | HexNAc(6)Hex(7)Fuc(3) |
| 75 | HexNAc(7)Hex(3)Fuc(1) |
| 76 | HexNAc(7)Hex(7)Fuc(1) |
| **Node** | **Glycan** |
| 77 | HexNAc(8)Hex(5)Fuc(1) |
| 78 | HexNAc(8)Hex(9)Fuc(1) |
| 79 | HexNAc(9)Hex(3)Fuc(1) |
| 80 | HexNAc(9)Hex(6)Fuc(1) |
| 81 | HexNAc(9)Hex(9)Fuc(1) |
| 82 | HexNAc(3)Hex(4)Fuc(1)NeuAc(1) |
| 83 | HexNAc(3)Hex(4)Fuc(2)NeuAc(1) |
| 84 | HexNAc(3)Hex(4)NeuAc(1) |
| 85 | HexNAc(3)Hex(5)Fuc(1)NeuAc(1) |
| 86 | HexNAc(3)Hex(5)NeuAc(1) |
| 87 | HexNAc(3)Hex(6)Fuc(1)NeuAc(1) |
| 88 | HexNAc(3)Hex(6)NeuAc(1) |
| 89 | HexNAc(4)Hex(3)NeuAc(1) |
| 90 | HexNAc(4)Hex(4)Fuc(1)NeuAc(1) |
| 91 | HexNAc(4)Hex(4)NeuAc(1) |
| 92 | HexNAc(4)Hex(5)Fuc(1)NeuAc(1) |
| 93 | HexNAc(4)Hex(5)Fuc(1)NeuAc(2) |
| 94 | HexNAc(4)Hex(5)Fuc(2)NeuAc(1) |
| 95 | HexNAc(4)Hex(5)Fuc(3)NeuAc(1) |
| 96 | HexNAc(4)Hex(5)NeuAc(1) |
| 97 | HexNAc(4)Hex(6)Fuc(1)NeuAc(1) |
| **Node** | **Glycan** |
| 98 | HexNAc(4)Hex(6)NeuAc(1) |
| 99 | HexNAc(4)Hex(7)NeuAc(1) |
| 100 | HexNAc(5)Hex(3)Fuc(1)NeuAc(1) |
| 101 | HexNAc(5)Hex(4)Fuc(1)NeuAc(1) |
| 102 | HexNAc(5)Hex(4)NeuAc(1) |
| 103 | HexNAc(5)Hex(4)NeuAc(2) |
| 104 | HexNAc(5)Hex(5)Fuc(1)NeuAc(2) |
| 105 | HexNAc(5)Hex(5)Fuc(2)NeuAc(1) |
| 106 | HexNAc(5)Hex(6)NeuAc(2) |
| 107 | HexNAc(6)Hex(3)Fuc(1)NeuAc(1) |
| 108 | HexNAc(6)Hex(3)Fuc(1)NeuAc(2) |
| 109 | HexNAc(6)Hex(5)Fuc(1)NeuAc(2) |
| 110 | HexNAc(6)Hex(5)Fuc(2)NeuAc(1) |
| 111 | HexNAc(6)Hex(6)Fuc(2)NeuAc(1) |
| 112 | HexNAc(6)Hex(6)NeuAc(1) |
| 113 | HexNAc(6)Hex(7)Fuc(3)NeuAc(1) |
| 114 | HexNAc(6)Hex(7)NeuAc(3) |
| 115 | HexNAc(6)Hex(7)NeuAc(4) |
| 116 | HexNAc(6)Hex(9)Fuc(1)NeuAc(2) |
| 117 | HexNAc(7)Hex(8)Fuc(1)NeuAc(1) |


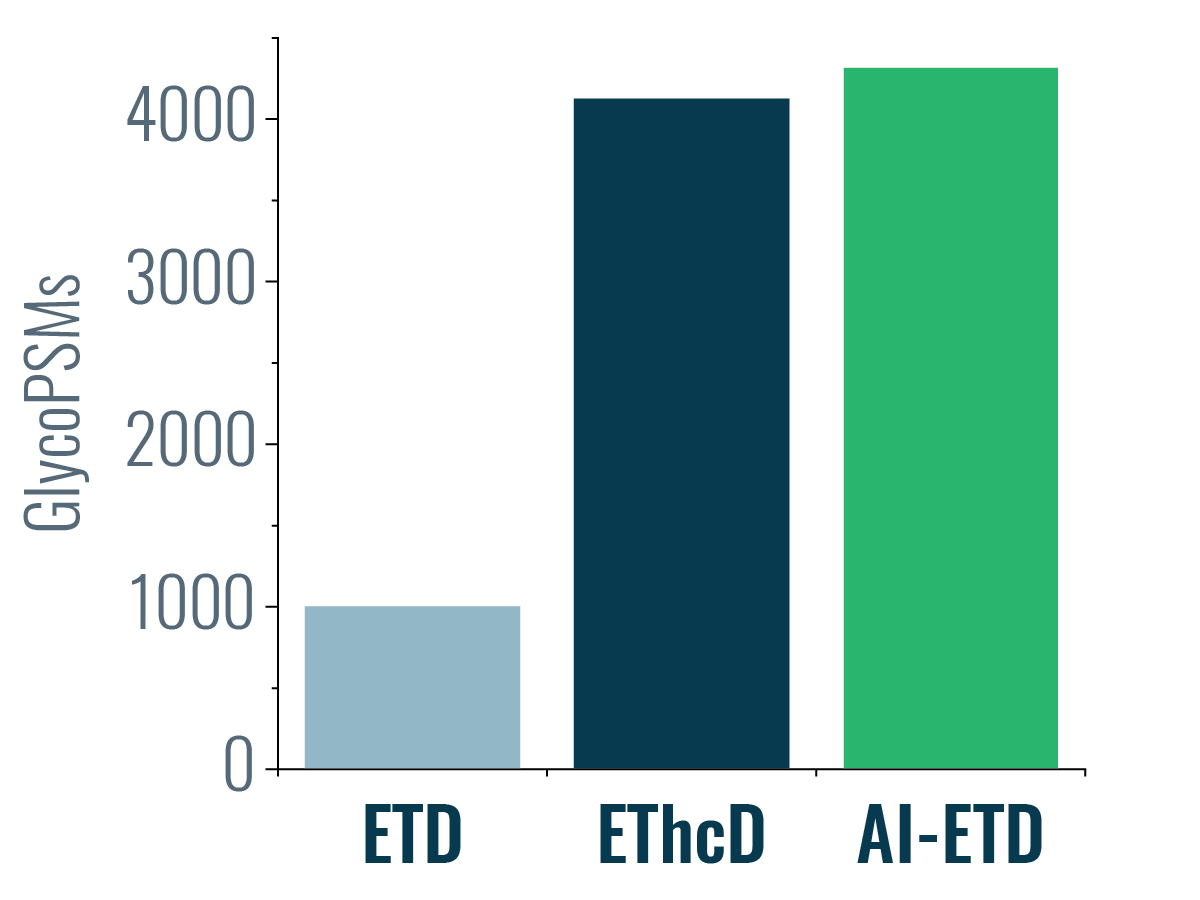


**Supplementary Figure 1. Comparing ETD, ETD with supplemental activation using higher energy collisional dissociation (EThcD) and AI-ETD.** Supplementary activation methods like EThcD and AI-ETD provide substantially more intact glycopeptide identifications than standard ETD, and AI-ETD provides a small boost in identifications over EThcD.


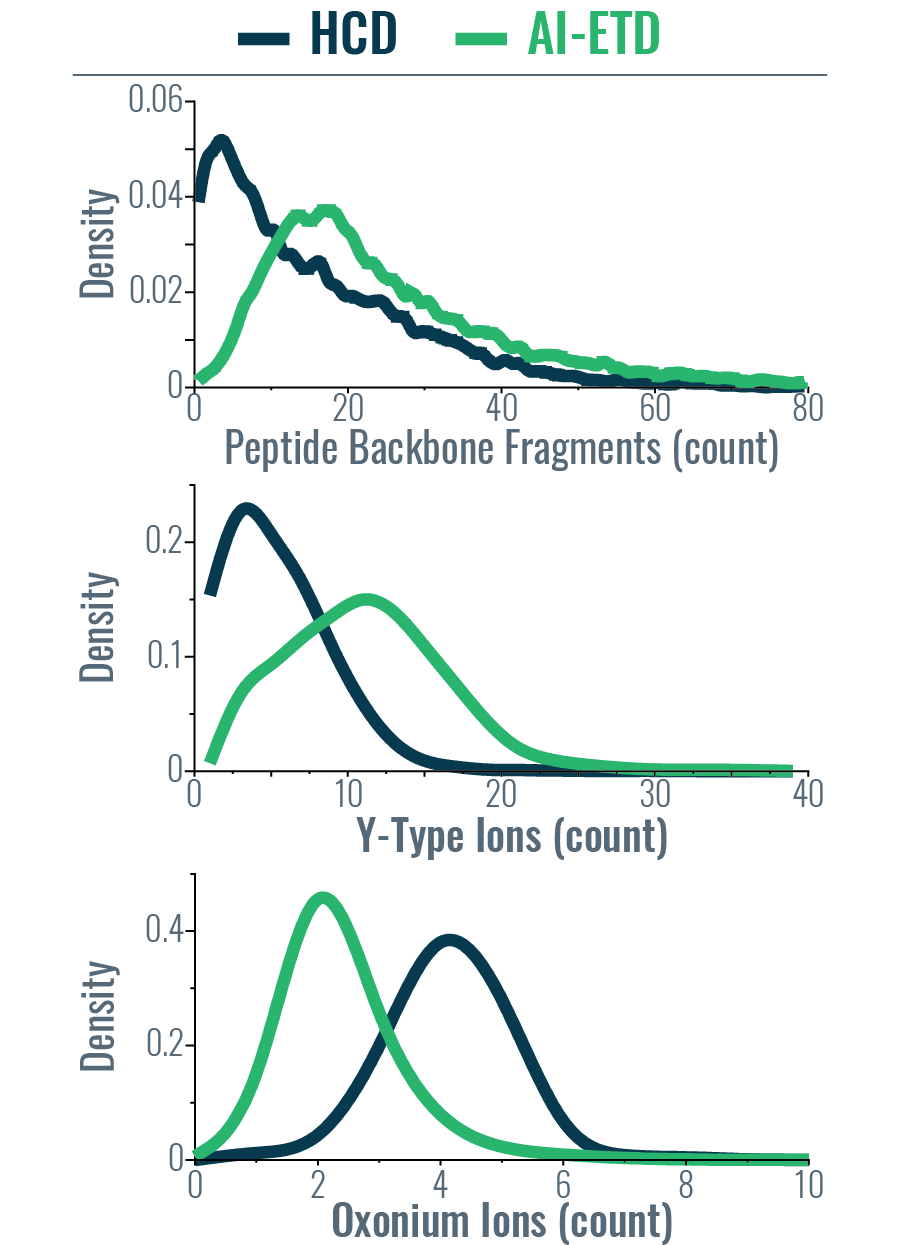


**Supplementary Figure 2. Distributions of fragment ions generated by AI-ETD (green) and HCD (dark blue).** Density plots show the number of peptide backbone fragments (top), Y-type ions (middle), and oxonium ions (bottom) produced by each fragmentation method.


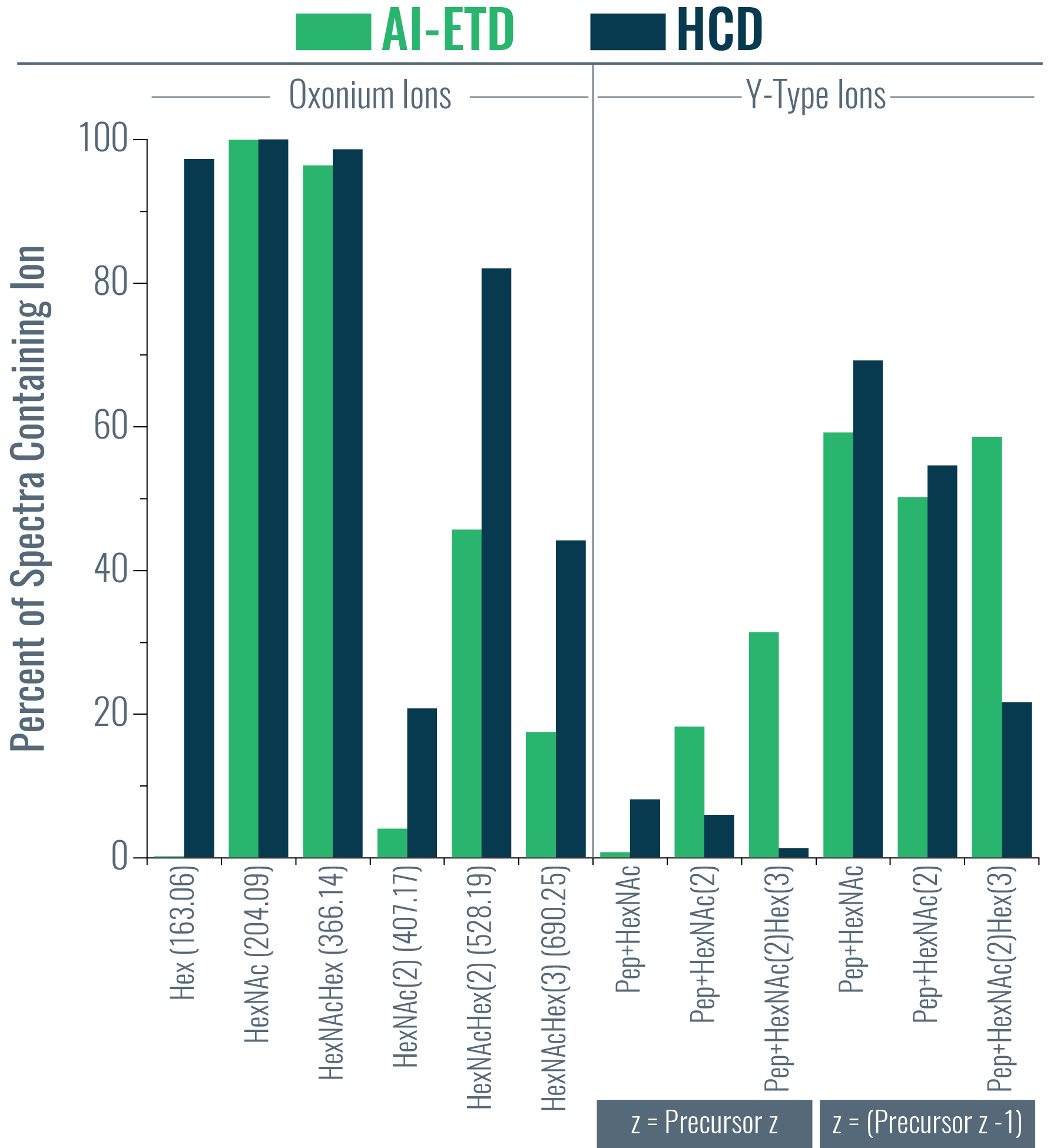


**Supplementary Figure 3. Percent of spectra containing specific oxonium and Y-type ions.** The bar graph provides the percent of glycoPSMs from AI-ETD (green) and HCD (dark blue) spectra containing specific oxonium and Y-type ion. For the Y-type ions, the left three ions have the same charge as the precursor ion, while the right three ions are for those Y-type ions that had one fewer charge than the precursor ion. This is indicated by the grey boxes below the x-axis, where *z* = charge.


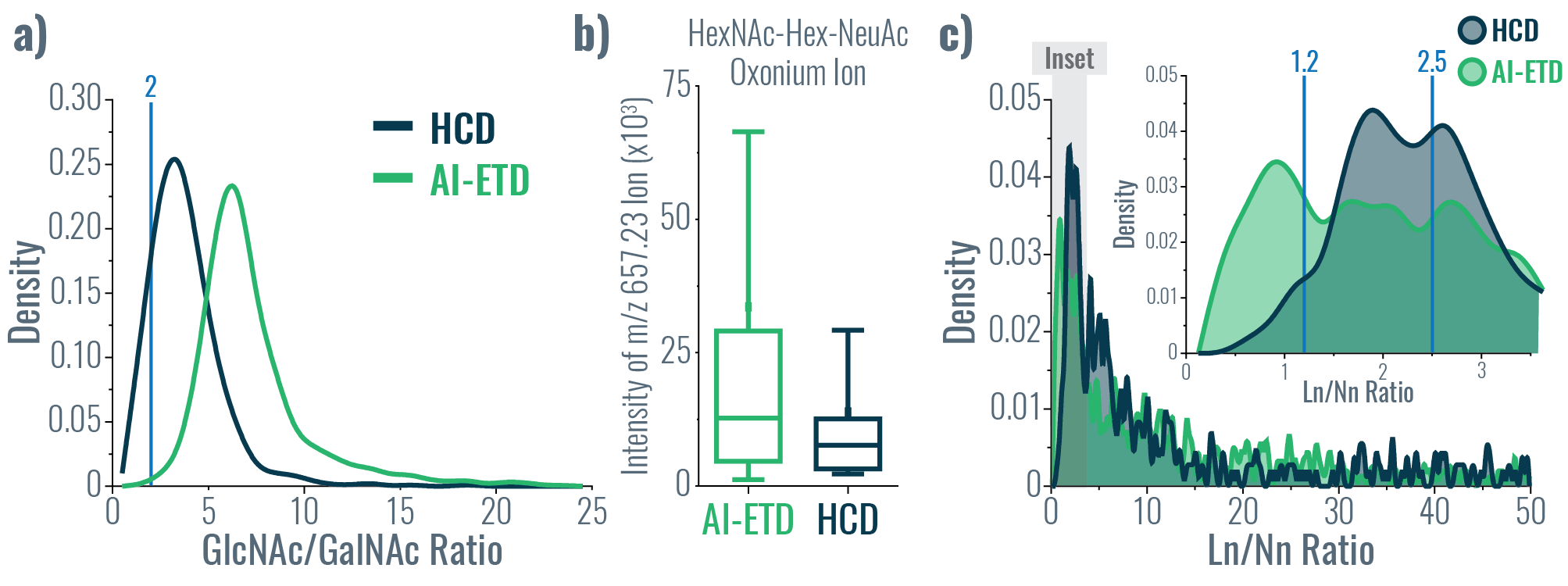


**Supplementary Figure 4. Using oxonium ions to differentiate glycan isomers.** **a)** GlcNAc/GalNAc ratios, as defined by Halim et al.^1^ and Yu et al.^2^, are calculated based on oxonium ion intensities to aid in defining HexNAc isomers as either GalNAc or GlcNAc. Ratios less than one indicate the presence of GalNAc residues, while ratios greater than two indicate the presence of GlcNAc residues (both cutoffs defined by vertical lines on the graph). The distributions of GlcNAc/GalNAc ratios calculated from oxonium ions from HCD and AI-ETD spectra are shown in dark blue and green, respectively. **b)** The box plot shows the distribution of intensities measured for the HexNAcHexNeuAc oxonium ion (m/z 657.2349) in AI-ETD and HCD glycoPSMs that contained a Neu5Ac residue. Median and quartile values are provided by the center line and box boundaries, respectively. Whisker show 10^th^ and 90^th^ percentiles. **c)** The Ln/Nn ratio, as defined by Pett et al., provides insight in the presence of either α2,3 and α2,6 linked NeuAc residues.^3^ The total distributions of Ln/Nn ratios are given for HCD (dark blue) and AI-ETD (green) spectra, with the inset showing a zoom on ratios less than 3.5 (the inset region is shown in grey on the larger graph). Vertical lines show approximate cutoffs for determining α2,3 (less than 1.2) versus α2,6 (greater than 2.5) linked NeuAc residues based on uncorrected Ln/Nn ratios provided by Pett et al.^3^


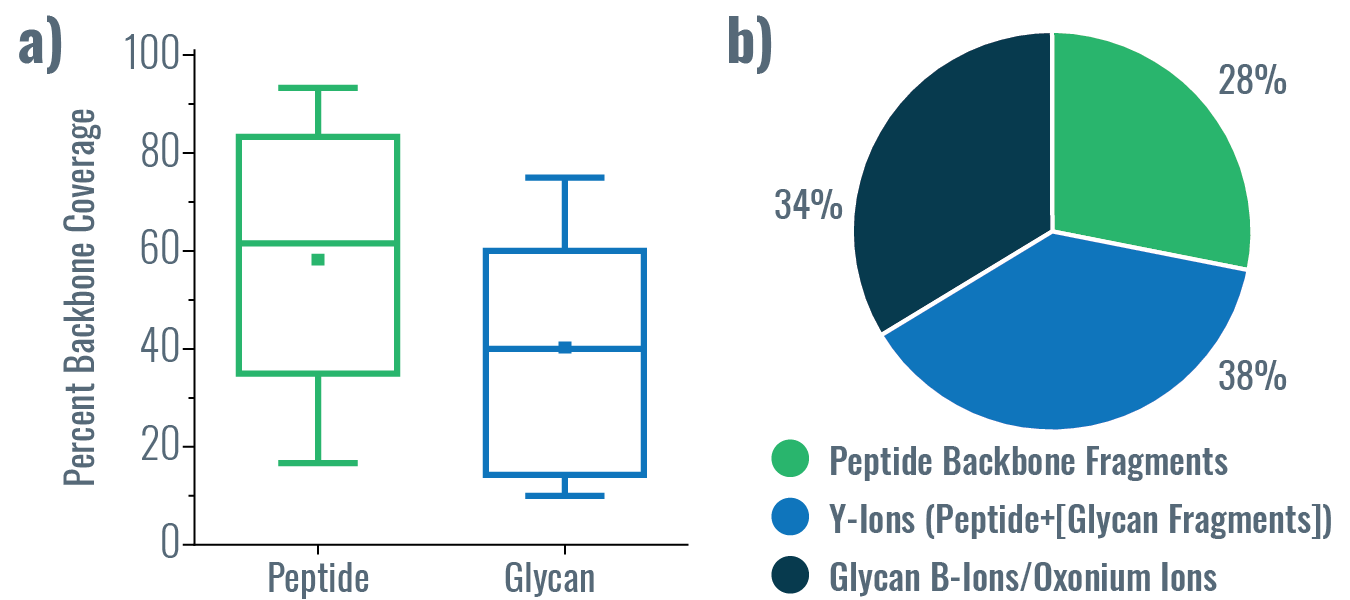


**Supplementary Figure 5. HCD performance characteristics. a)** Distribution of percent peptide backbone coverage and glycan coverage seen in HCD spectra. Median and quartile values are provided by the center line and box boundaries, respectively. Whisker show 10^th^ and 90^th^ percentiles, and the small square indicates the average. **b)** Average percent of explained ion current in product ions in HCD spectra from peptide backbone cleavage fragments, Y-ions (i.e., intact peptide sequence with fragments of the glycan moiety), and B-ions/oxonium ions.


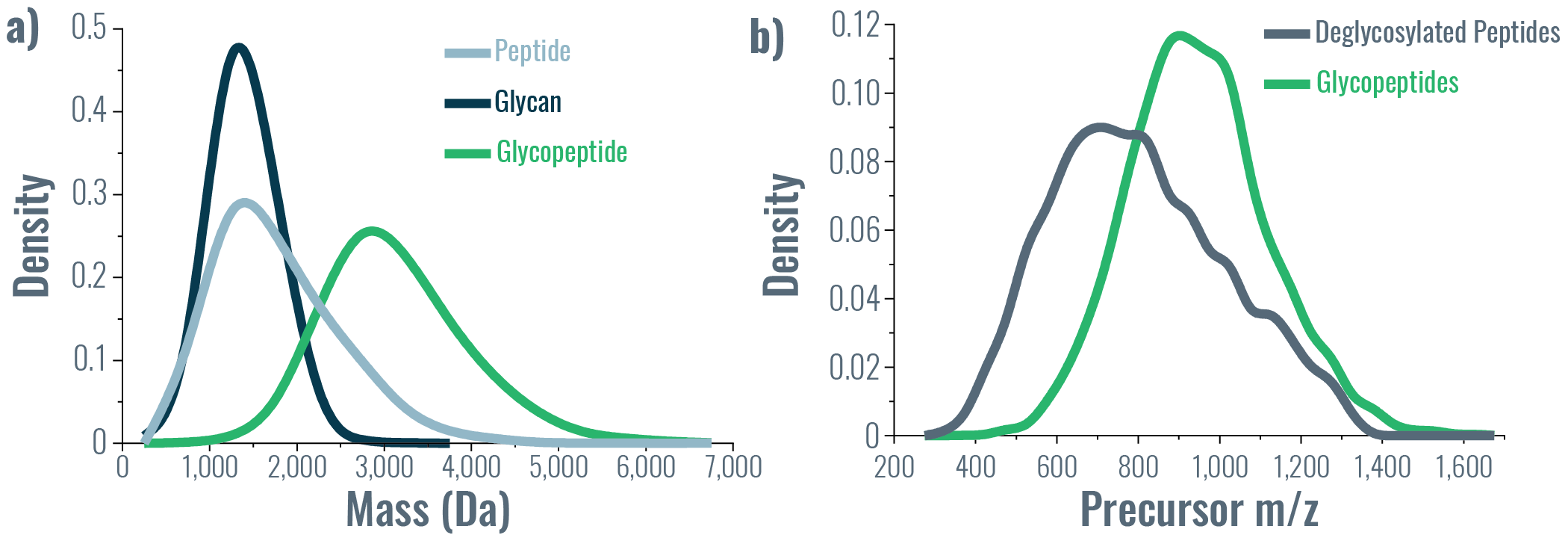


**Supplementary Figure 6. Mass and m/z distributions of glycoproteomics data.** **a)** Density plots show the mass distributions of intact glycopeptides (green), peptide sequences from glycopeptides without the glycan mass considered (light blue), and glycans from glycopeptides with the peptide mass considered (dark blue). **b)** Density plots provide the precursor *m/z* distribution from deglycosylated peptides, i.e., from the PNGaseF treated samples (grey) and the intact glycopeptides (green), highlighting the shift to significantly higher precursor *m/z* values in experiments analyzing intact glycopeptides.


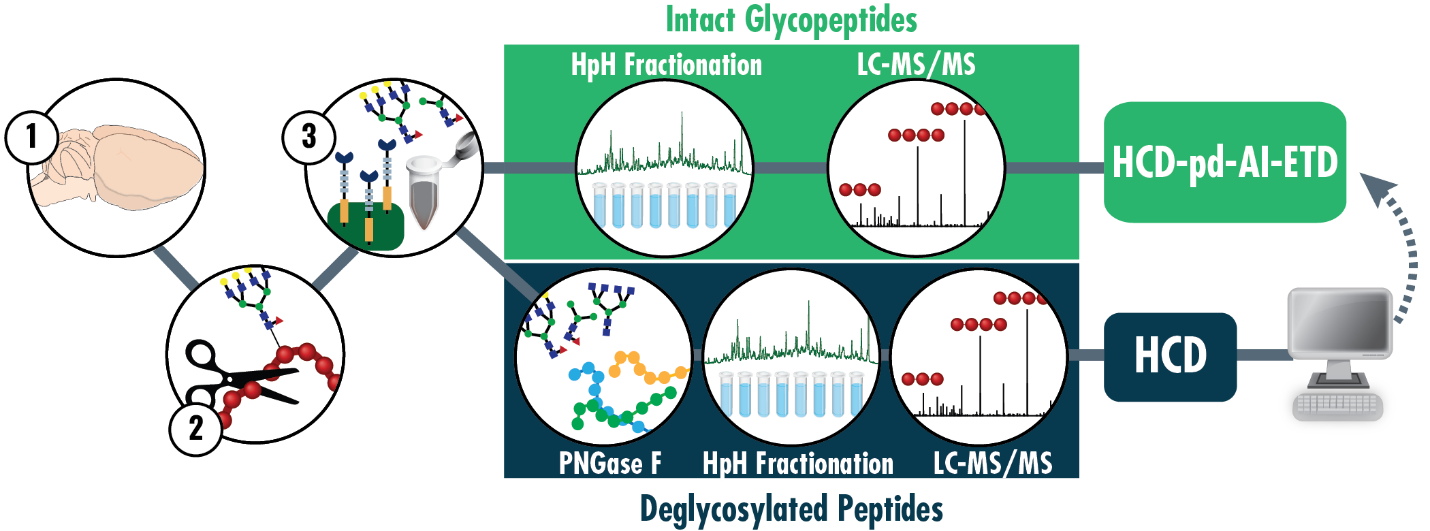


**Supplementary Figure 7. Workflow for in-depth profiling of the glycoproteome via intact glycopeptides with AI-ETD.** Mouse brains are (1) lysed, (2) digested with trypsin, and (3) enriched using a lectin-spin column format. A portion of enriched glycopeptides are deglycosylated with PNGaseF, fractionated using high pH (HpH) reversed phase separations, and analyzed to generate a glycoprotein database to search intact glycopeptide data. The majority of the enriched glycopeptides are kept as intact species, fractionated with HpH separations, and analyzed with LC-MS/MS using a higher-energy collision dissociation-product dependent-activated ion electron transfer dissociation (HCD-pd-AI-ETD) acquisition method.


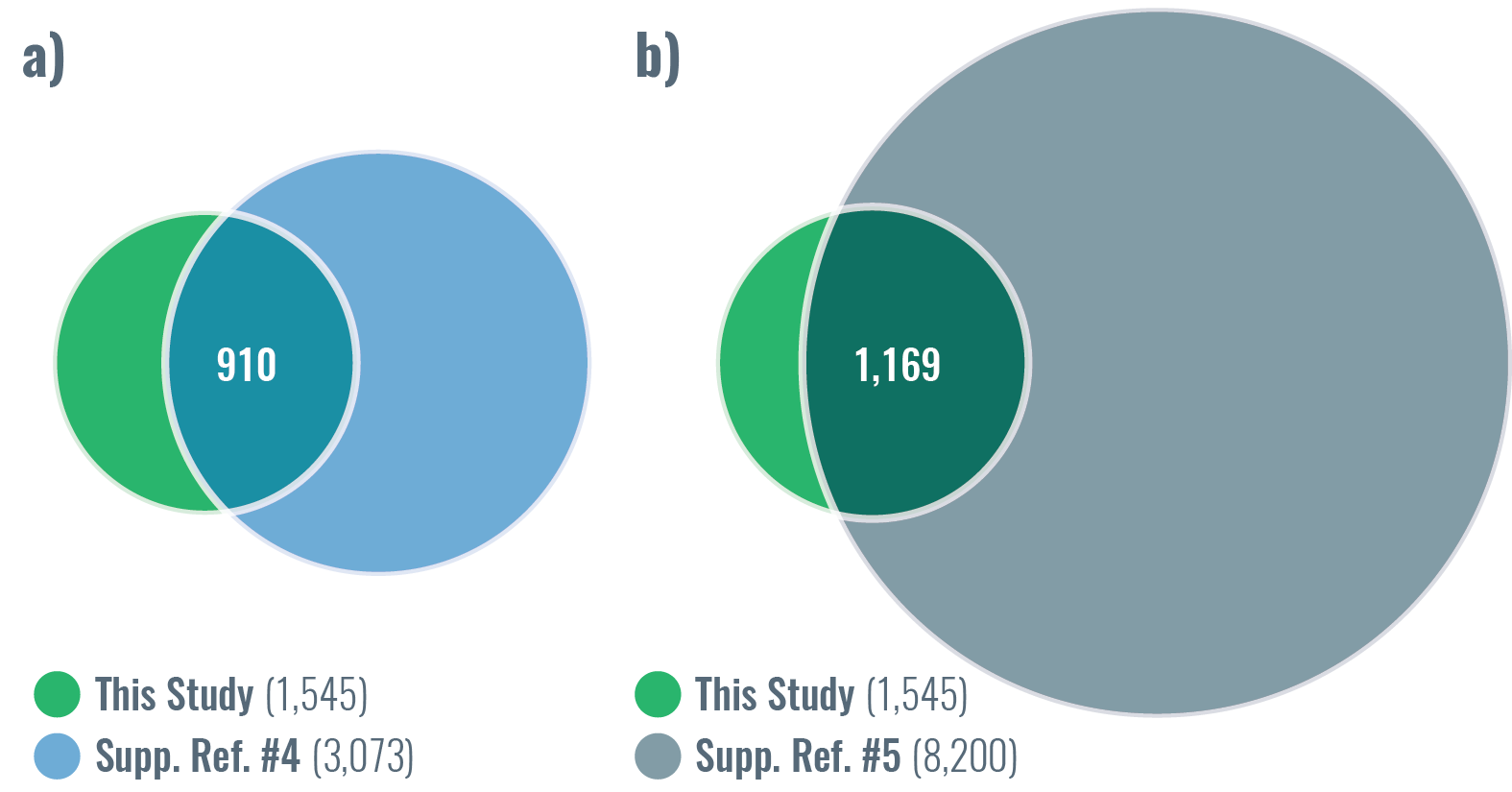


**Supplementary Figure 8. Comparison of glycosites to large-scale deglycoproteomic experiments.** The results of this study were compared with the data from Zielinska et al.^4^ **(a)** and Fang et al.^5^ **(b).** Both other studies used PNGaseF to removed N-glycans and do large-scale sequencing of the deglycoproteome to capture sites of N-glycosylation.^4,5^ Venn diagrams show the overlap in N-glycosites characterized in our intact glycopeptide data and their deglycoproteomic data. Note, Zielinska et al. used trypsin and GluC as proteases and lectins to enrich, while Fang et al. used seven proteases and four different enrichment strategies.


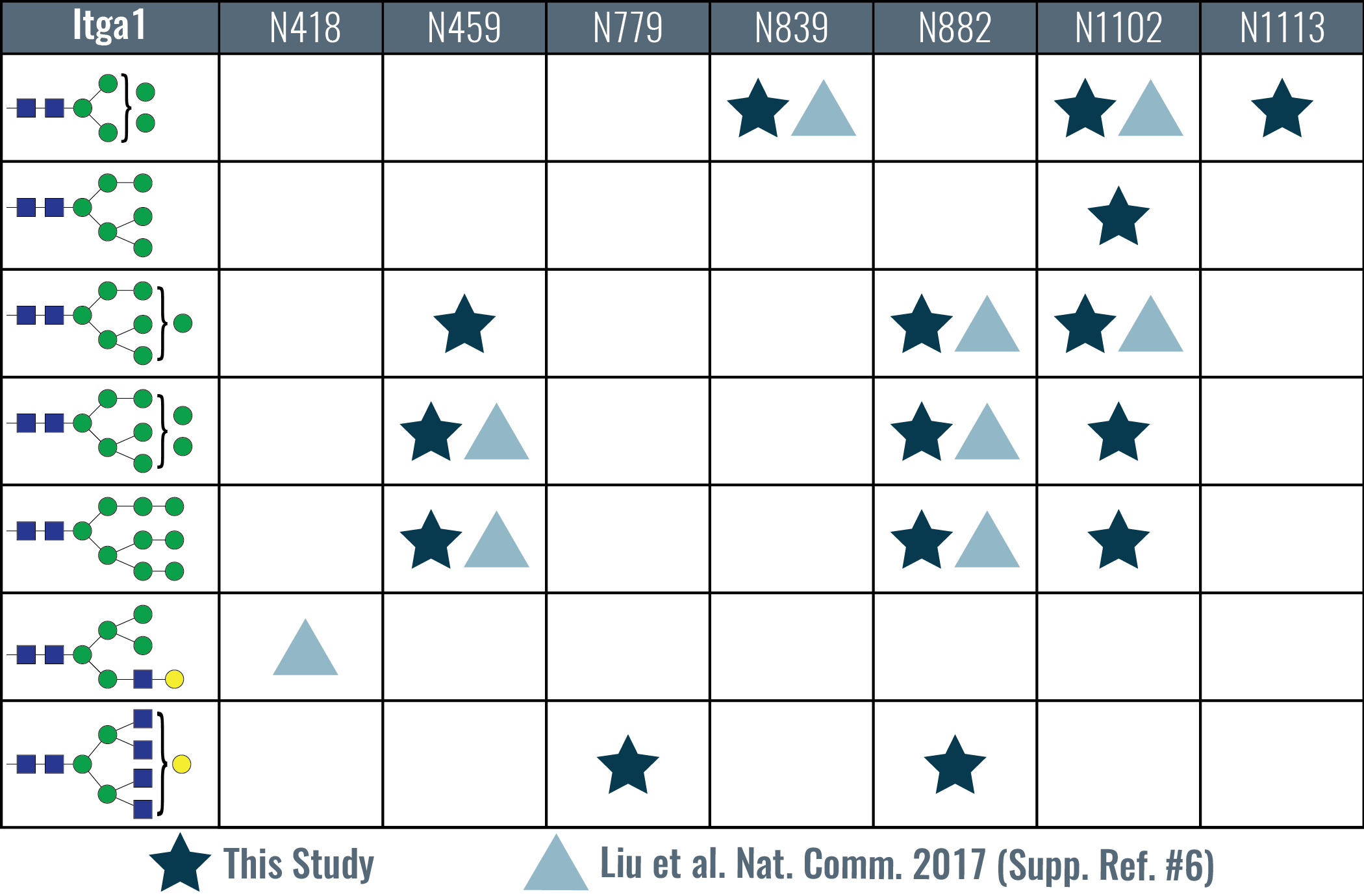


**Supplementary Figure 9. N-glycosites characterized in integrin alpha-1 (GN: Itga1, UniProt: Q3V3R4) in mouse brain.** Identified N-glycosites are listed in the top row of the table and glycans are in the leftmost column. Dark blue stars indicate glycosite/glycan combinations identified in this study and light blue triangles show identifications from Liu et al. (adapted from the table in Figure 8 of that manuscript).^6^ Eight of the nine combinations from the Liu et al. study are seen in this dataset, in addition to seven novel ones (including two novel glycosites).


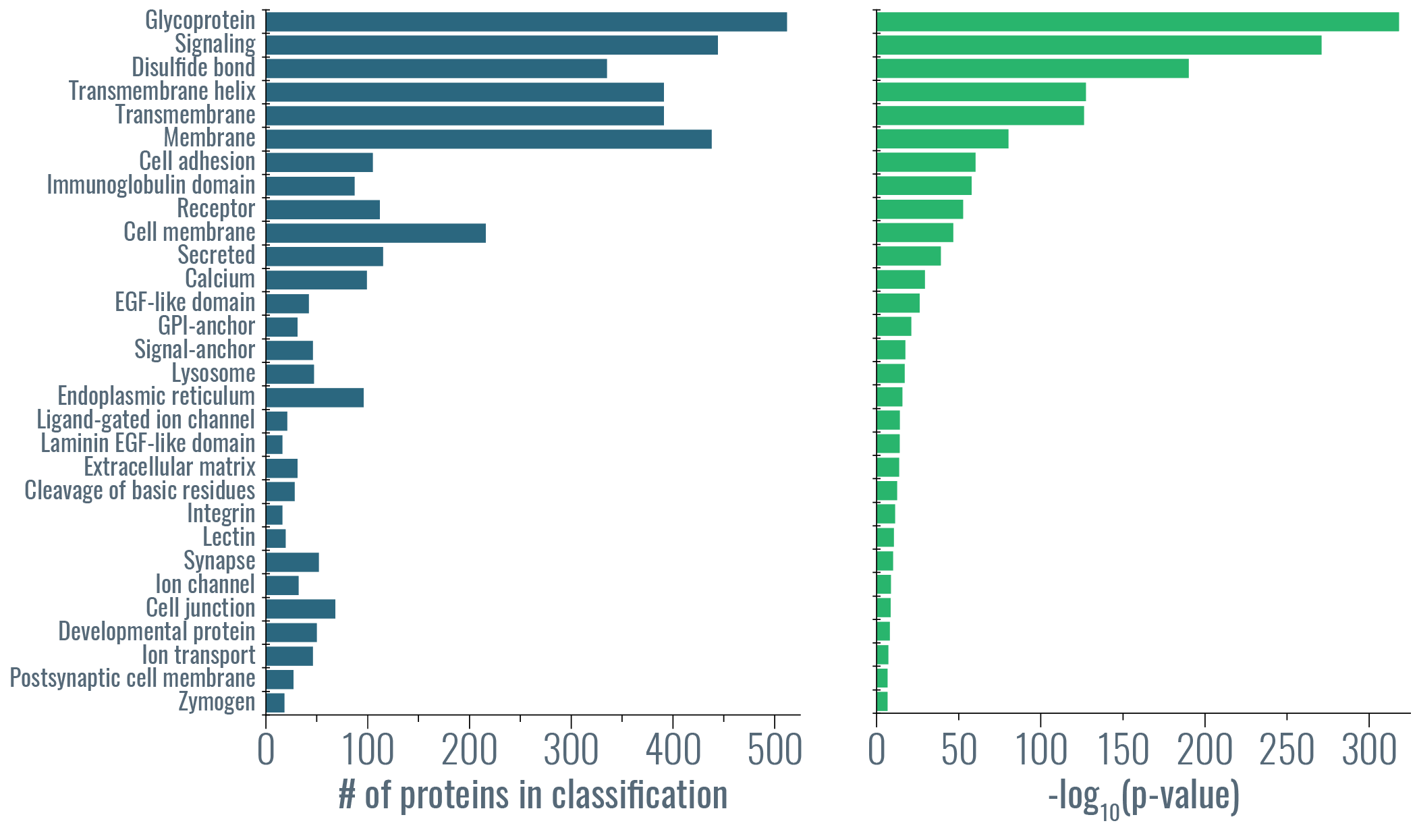


**Supplementary Figure 10.** **Gene ontology functional categories enriched terms for identified glycoproteins.** Gene ontology categories for the most enriched terms showing the number of proteins (dark blue) and the significance of the en­­richment (green) for each category.


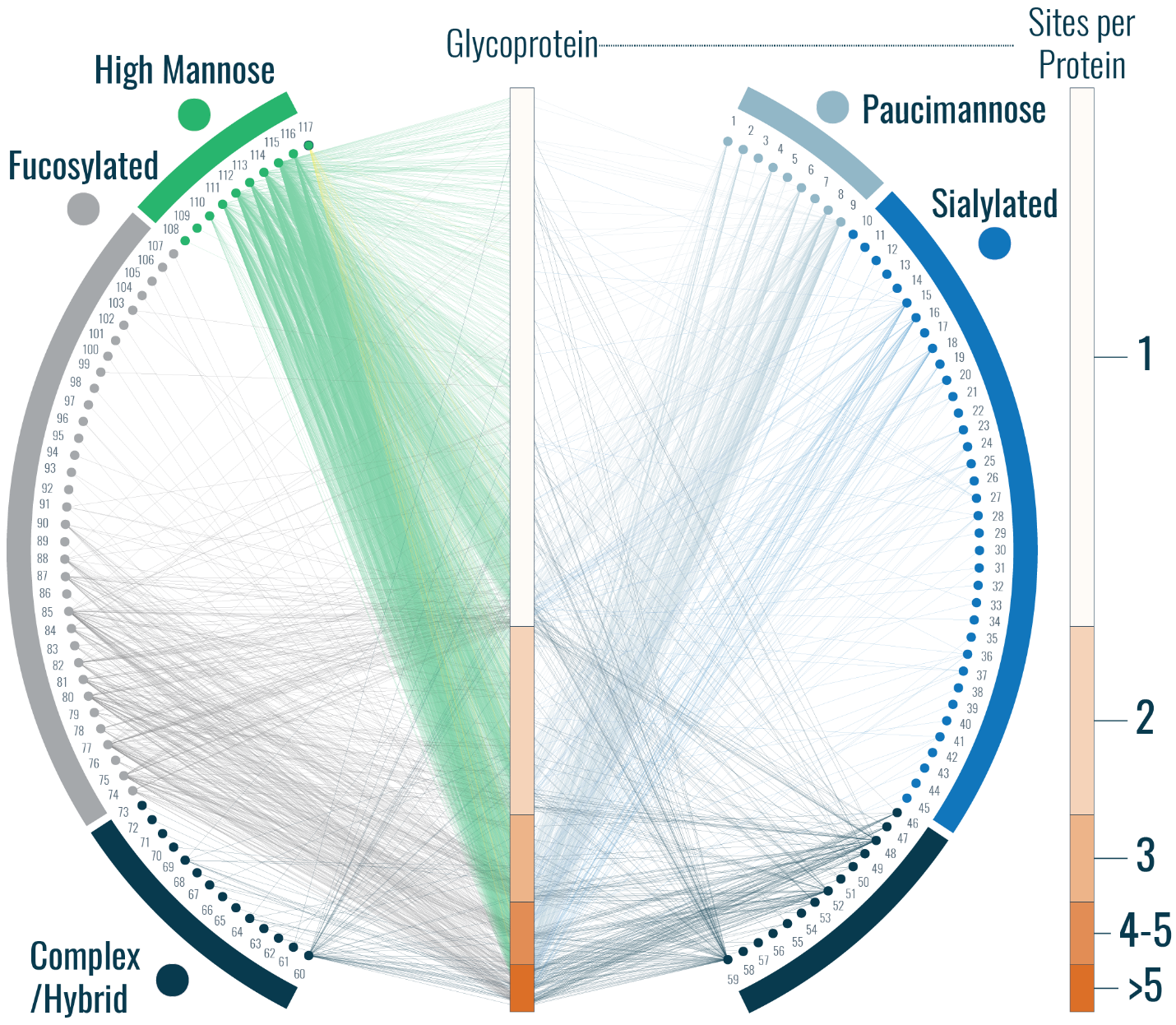


**Supplementary Figure 11. A larger version of the glycoprotein-glycan map in Figure 2f.** A glycoprotein-glycan network maps which glycans (outer circle, 117 total) modify which proteins (inner bar, 771 total). Glycoproteins are sorted by number of glycosites (scale to the right). Glycans are organized by classification, and edges are colored by the glycan node from which they originate, except for mannose-6-phosphate which has yellow edges. Glycan identities corresponding to node labels are given **Supplementary Table 1**. Note, paucimannose and sialylated glycans that contained fucose moieties are classified as paucimannose or sialylated instead of as fucosylated.


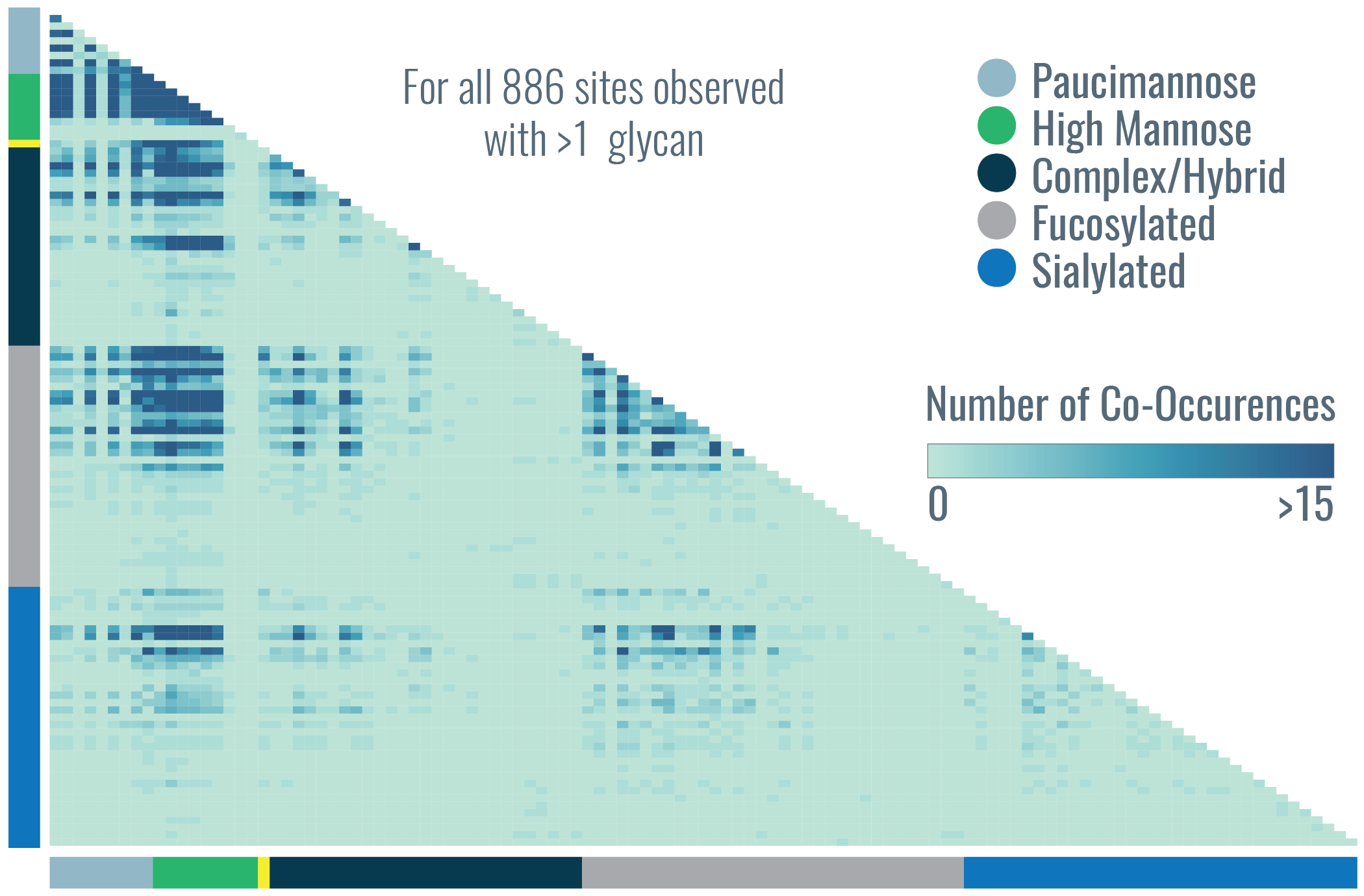


**Supplementary Figure 12. A larger version of the co-occurrence heat map in Figure 2g.** A heat map represents the number of times glycan pairs appeared together at the same glycosite, indicating which glycans contribute most to microheterogeneity of the >880 sites that had more than one glycan modifying them. Glycans are grouped together by type, as indicated in the key at the top right. Glycan identities are provided by order number in **Supplementary Table 2**.


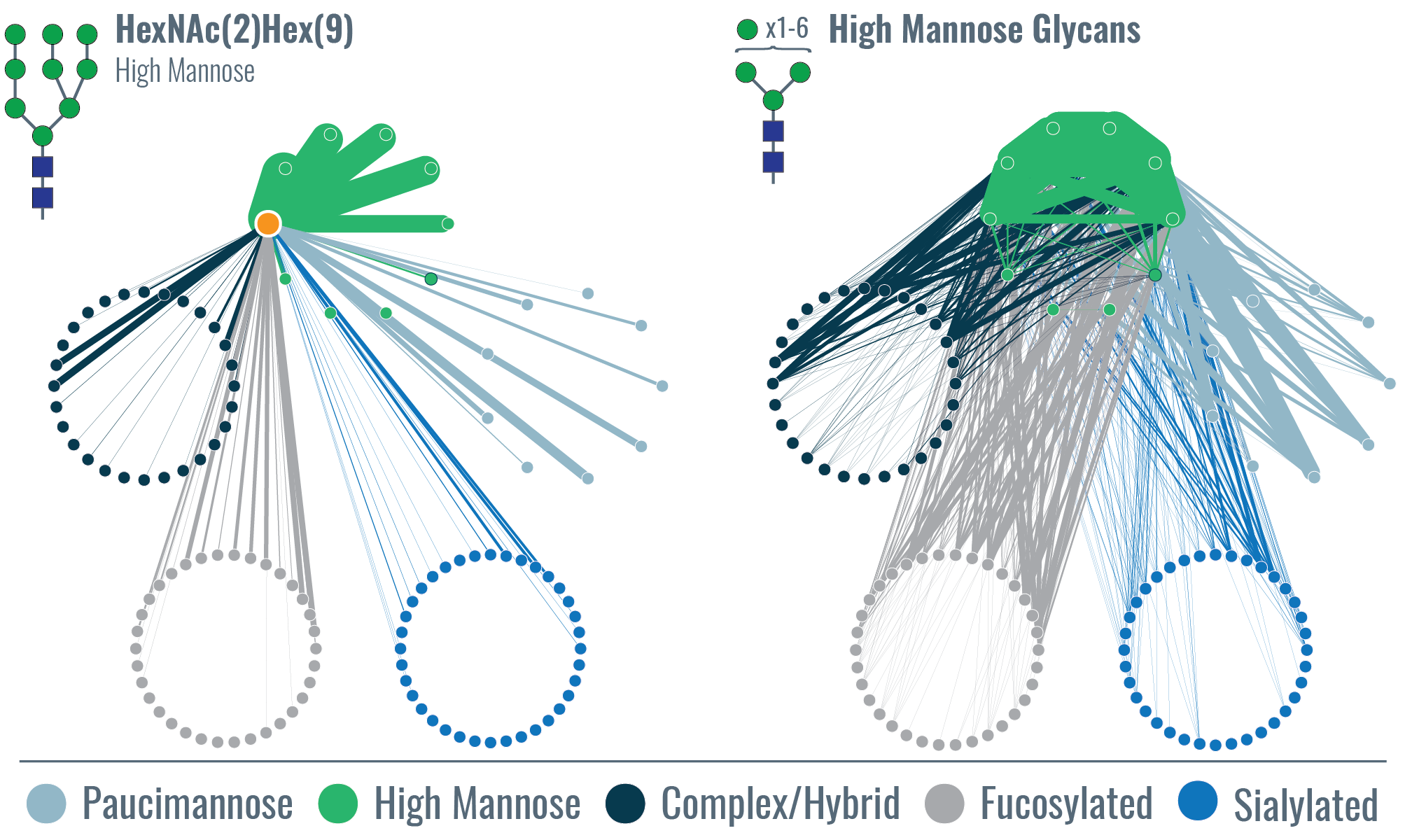


**Supplementary Figure 13. Glycan co-occurrence networks for a specific glycan and a class of glycans.** Glycan co-occurrence networks can show (left) all the glycans that co-occurred with the specific high mannose glycan HexNAc(2)Hex(9) (highlighted as an orange node, i.e., the source node), with the relative number of occurrences indicated by edge thickness. Edge color indicates the target node. They can also be used to visualize co-occurrence for more than one glycan or entire classes of glycans, in this case shown for all high mannose glycans. Interesting structure arises in the all high mannose glycan co-occurrence network. For example, co-occurrence of certain sialylated glycans (glycans #87-90) with several high mannose glycans is observed while others, e.g., glycans #109-113, occur with no high mannose glycans. Other such trends can be delineated amongst the glycan types that co-occur with high mannose glycans. The organization of the glycan co-occurrence network is given in **Figure 3a**, where glycans are sorted into larger circles based on glycan type, each node is one of the 117 glycans identified, and the numbers indicate glycans identities given in **Supplementary Table 3**.


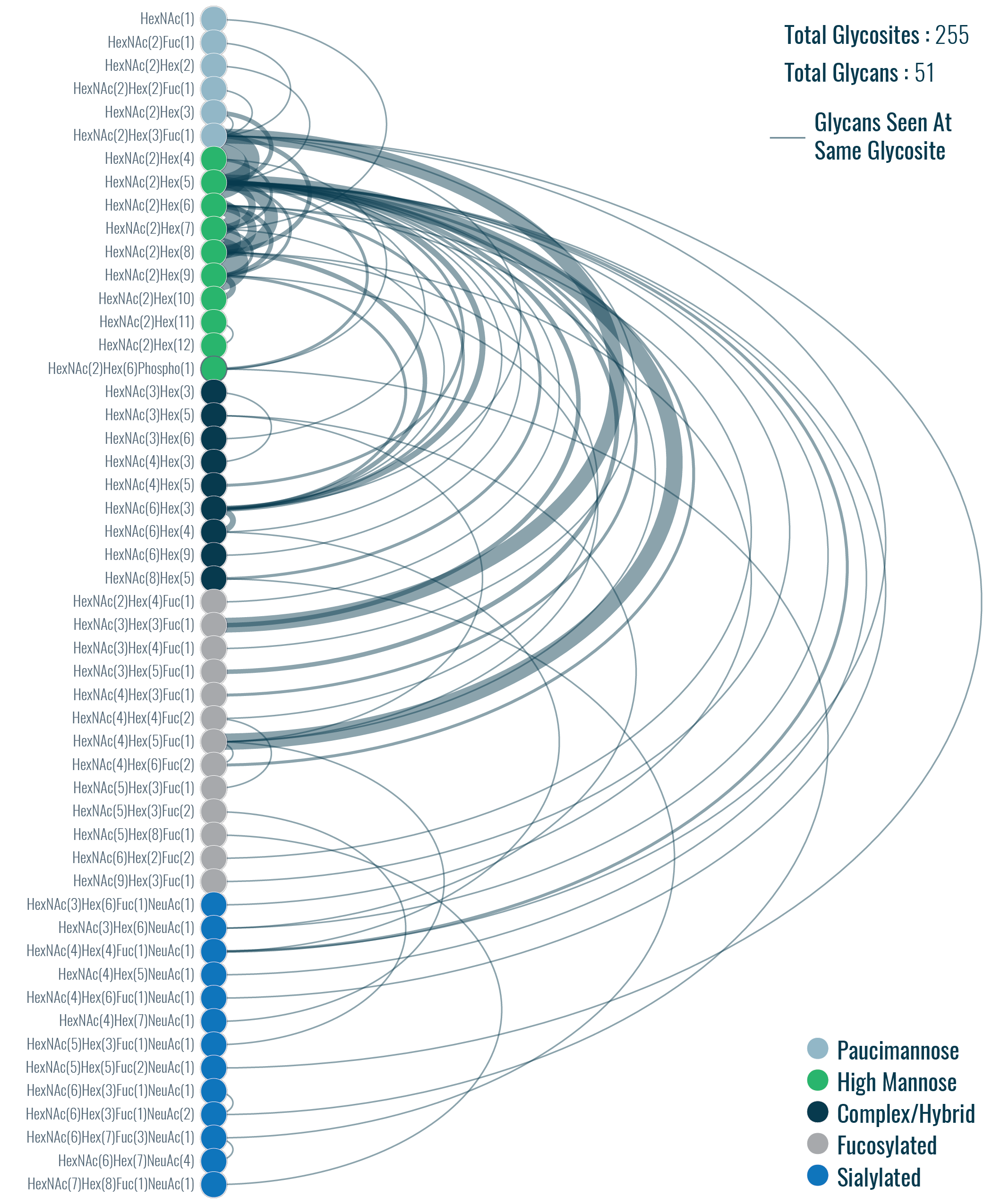


**Supplementary Figure 14. Arc plot representing glycan microheterogeneity for glycosites with two glycans per site.** Nodes are glycans (colors indicate glycan classification), and edges connect glycans that were seen at the same glycosite (weight indicates number of co-occurrences). Note, paucimannose and sialylated glycans that contain fucose moieties are classified as paucimannose or sialylated instead of as fucosylated.


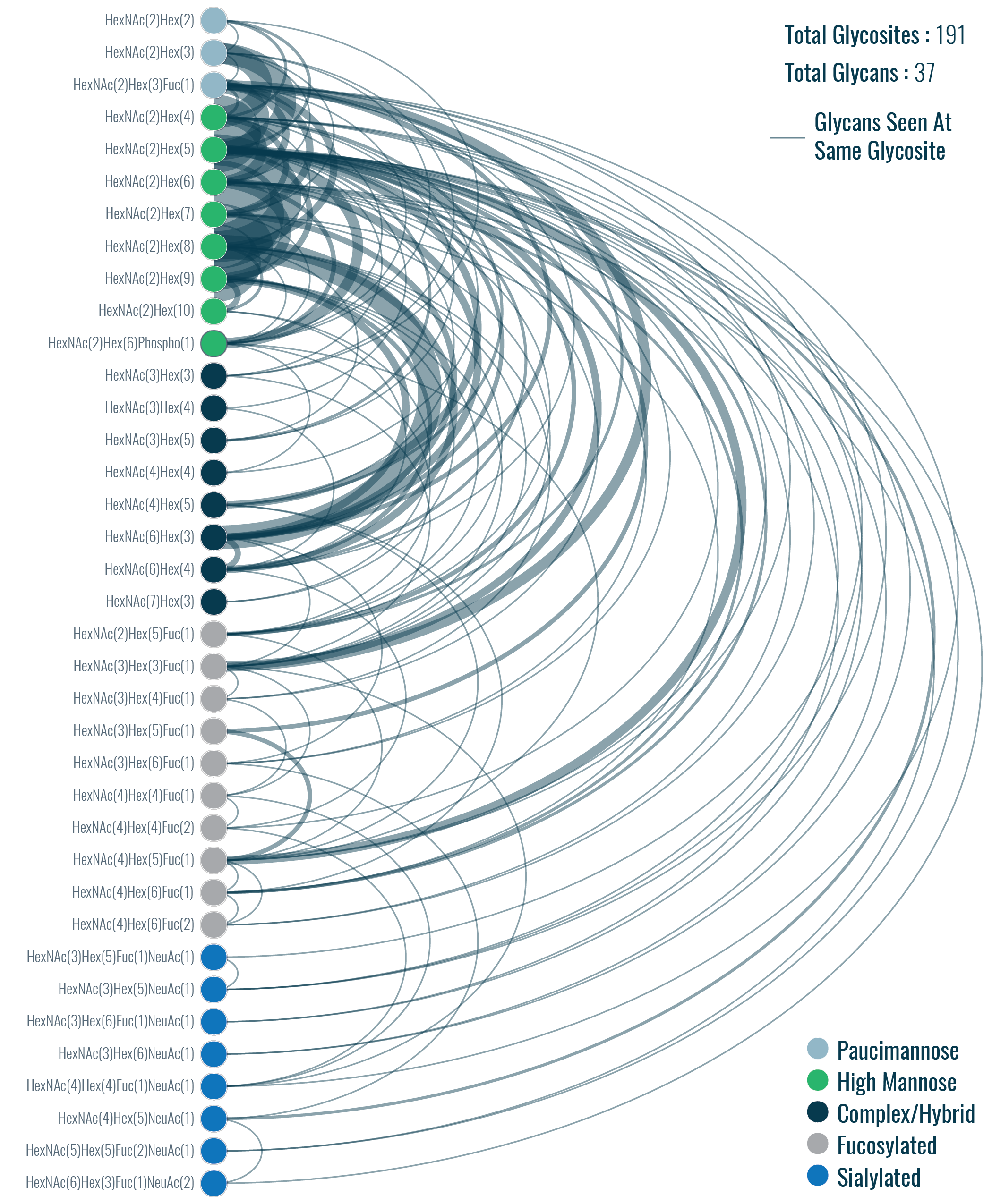


**Supplementary Figure 15. Arc plot representing glycan microheterogeneity for glycosites with three glycans per site.** Nodes are glycans (colors indicate glycan classification), and edges connect glycans that were seen at the same glycosite (weight indicates number of co-occurrences). Note, paucimannose and sialylated glycans that contain fucose moieties are classified as paucimannose or sialylated instead of as fucosylated.


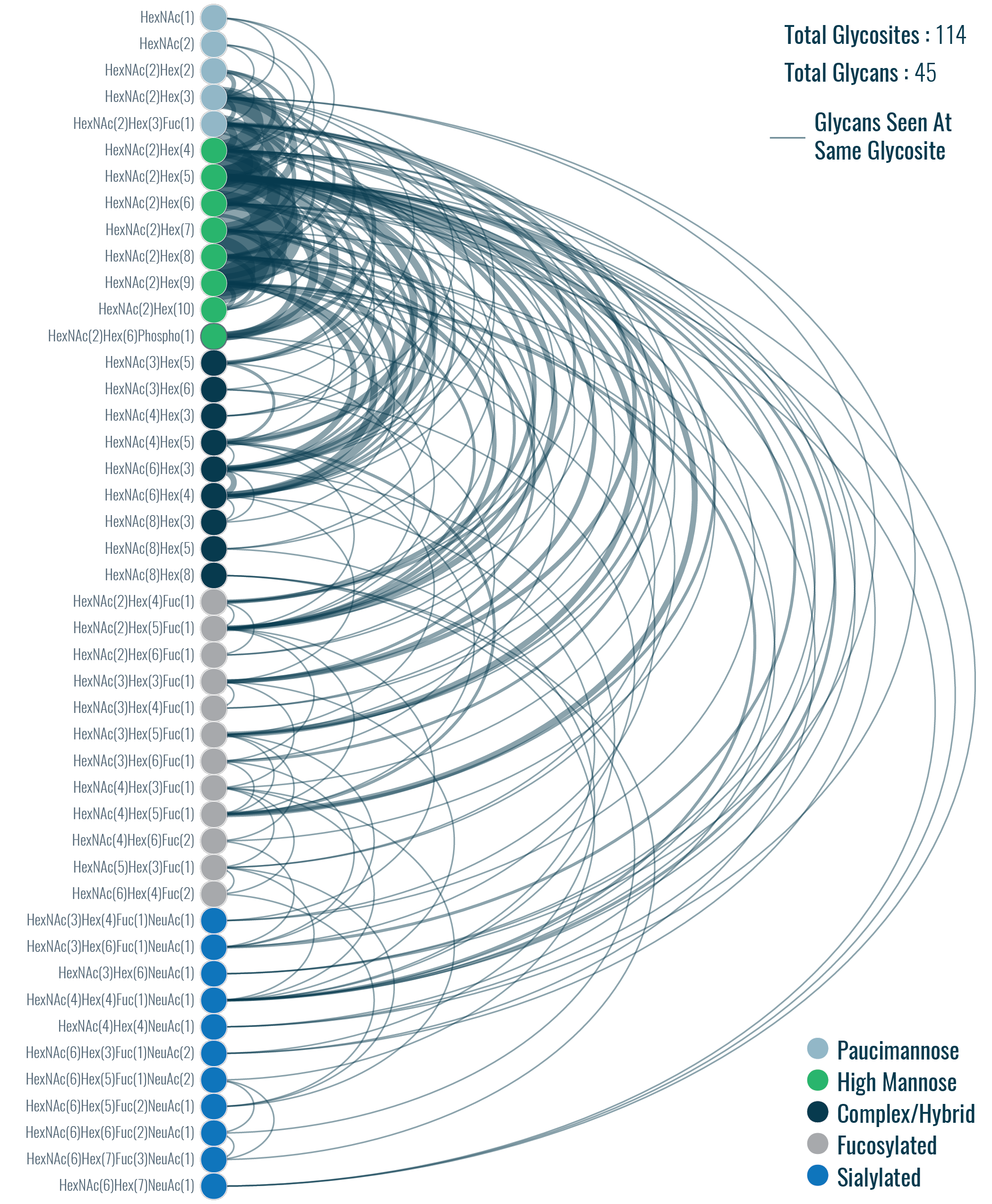


**Supplementary Figure 16. Arc plot representing glycan microheterogeneity for glycosites with four glycans per site.** Nodes are glycans (colors indicate glycan classification), edges connect glycans that were seen at the same glycosite (weight indicates number of co-occurrences). Note, paucimannose and sialylated glycans that contain fucose moieties are classified as paucimannose or sialylated instead of as fucosylated.


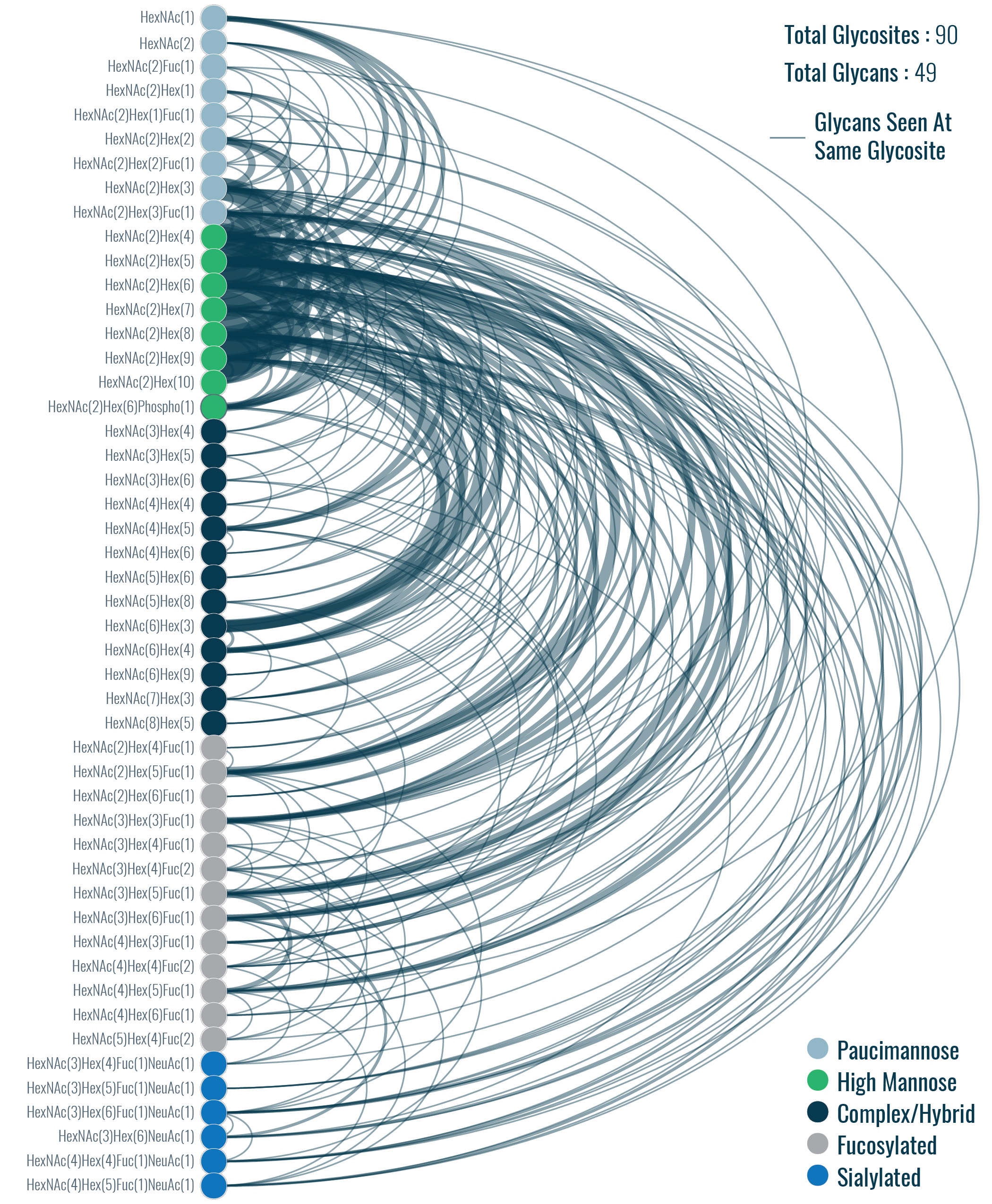


**Supplementary Figure 17. Arc plot representing glycan microheterogeneity for glycosites with five glycans per site.** Nodes are glycans (colors indicate glycan classification), and edges connect glycans that were seen at the same glycosite (weight indicates number of co-occurrences). Note, paucimannose and sialylated glycans that contain fucose moieties are classified as paucimannose or sialylated instead of as fucosylated.


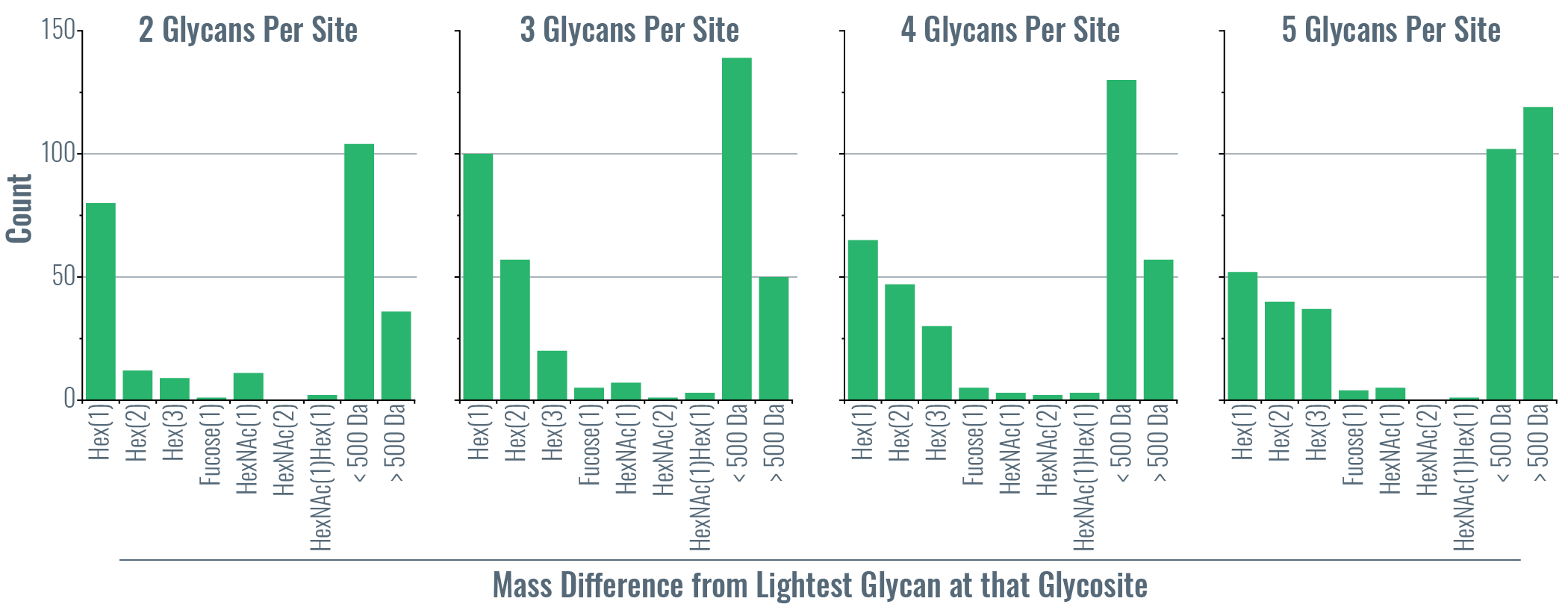


**Supplementary Figure 18. Mass differences between glycans that occupy the same glycosite.** A distribution of mass differences relative to the lightest glycan identified are plotted for all glycans seen at a given glycosite for glycosites with two, three, four, and five distinct glycans. Common mass differences for glycan are shown on the x-axis. For example, if both HexNAc(2)Hex(8) and HexNAc(2)Hex(9) were identified at a glycosite that had two distinct glycans, that would contribute to a count of one to the Hex(1), i.e., one mannose residue, mass difference in the leftmost plot. Observing HexNAc(6)Hex(3) and HexNAc(6)Hex(4), i.e., a difference of one galactose residue, at the same glycosite would contribute to the same Hex(1) count. This provides a crude summary of glycan microheterogeneity at sites with multiple glycans, but discerning all patterns is difficult, especially for >2 glycans per site, because mass difference are all relative to the lightest glycan. For example, a glycan with three glycans HexNAc(2)Hex(8), HexNAc(6)Hex(3), and HexNAc(2)Hex(9) would give mass differences of ~2 Da and ~162 Da (i.e., Hex(1)). This shows a clear difference in a pair of glycans differing by one hexose residue, but the ~2 Da mass difference does not give clear insight into the relationship between the two high mannose glycans [HexNAc(2)Hex(8), HexNAc(2)Hex(9)] and the complex glycan [HexNAc(6)Hex(3)].


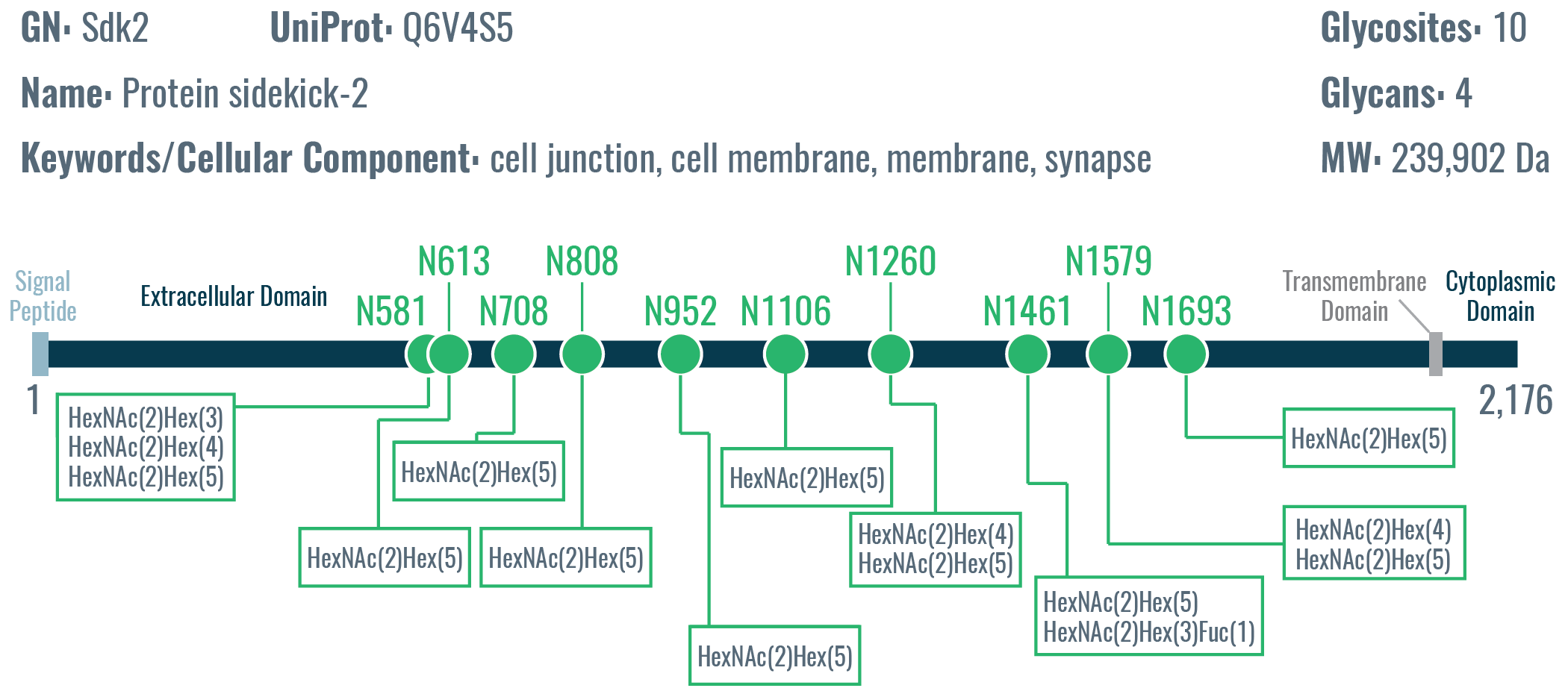


**Supplementary Figure 19. Protein sidekick-2: relatively high number of glycosites with relatively low glycan microheterogeneity.** Sdk2 is a somewhat large single-pass membrane glycoprotein found at the cell membrane that functions in cell adhesion, synapse assembly, and formation of neural circuits that detect motion. Even with ten glycosites identified, only four different glycans were characterized, which are smaller high mannose and fucosylated paucimannose core structure. Six of the ten sites had only one glycan, which was HexNAc(2)Hex(5) in all six cases.


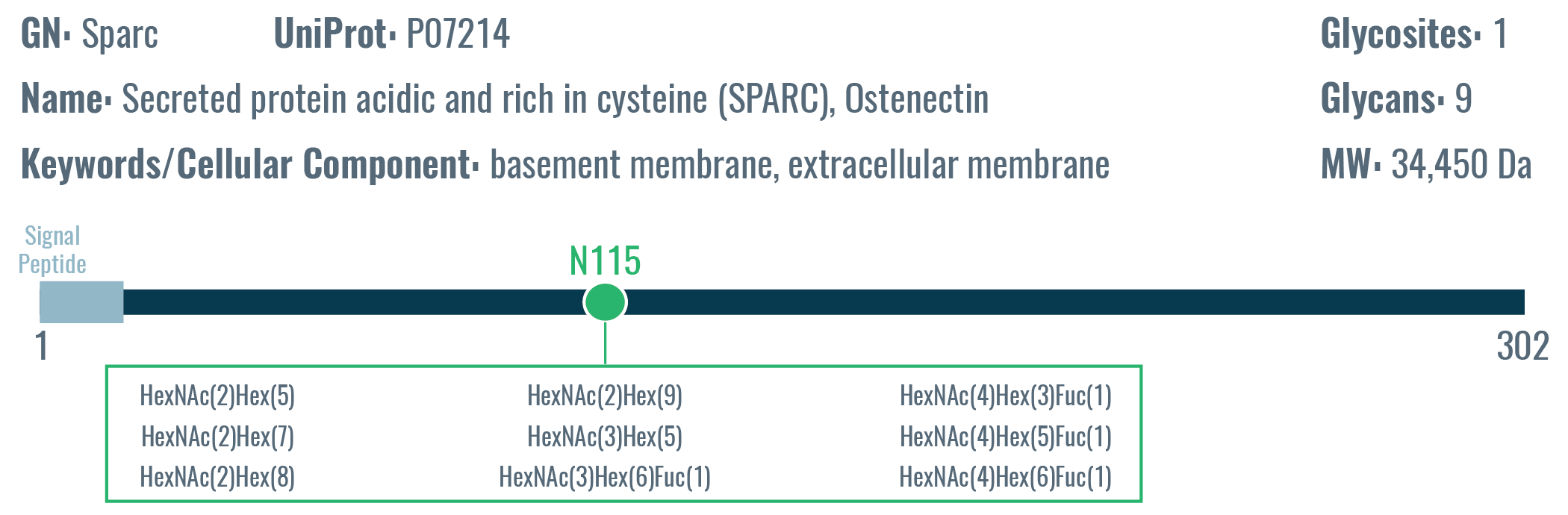


**Supplementary Figure 20. Secreted protein acidic and rich in cysteine (SPARC): only one glycosite with moderate glycan microheterogeneity.** Sparc is an extracellular membrane protein with only one known glycosite but nine different glycans were observed at that site. The degree of glycan heterogeneity is moderate, with high mannose, complex, and fucosylated glycans.


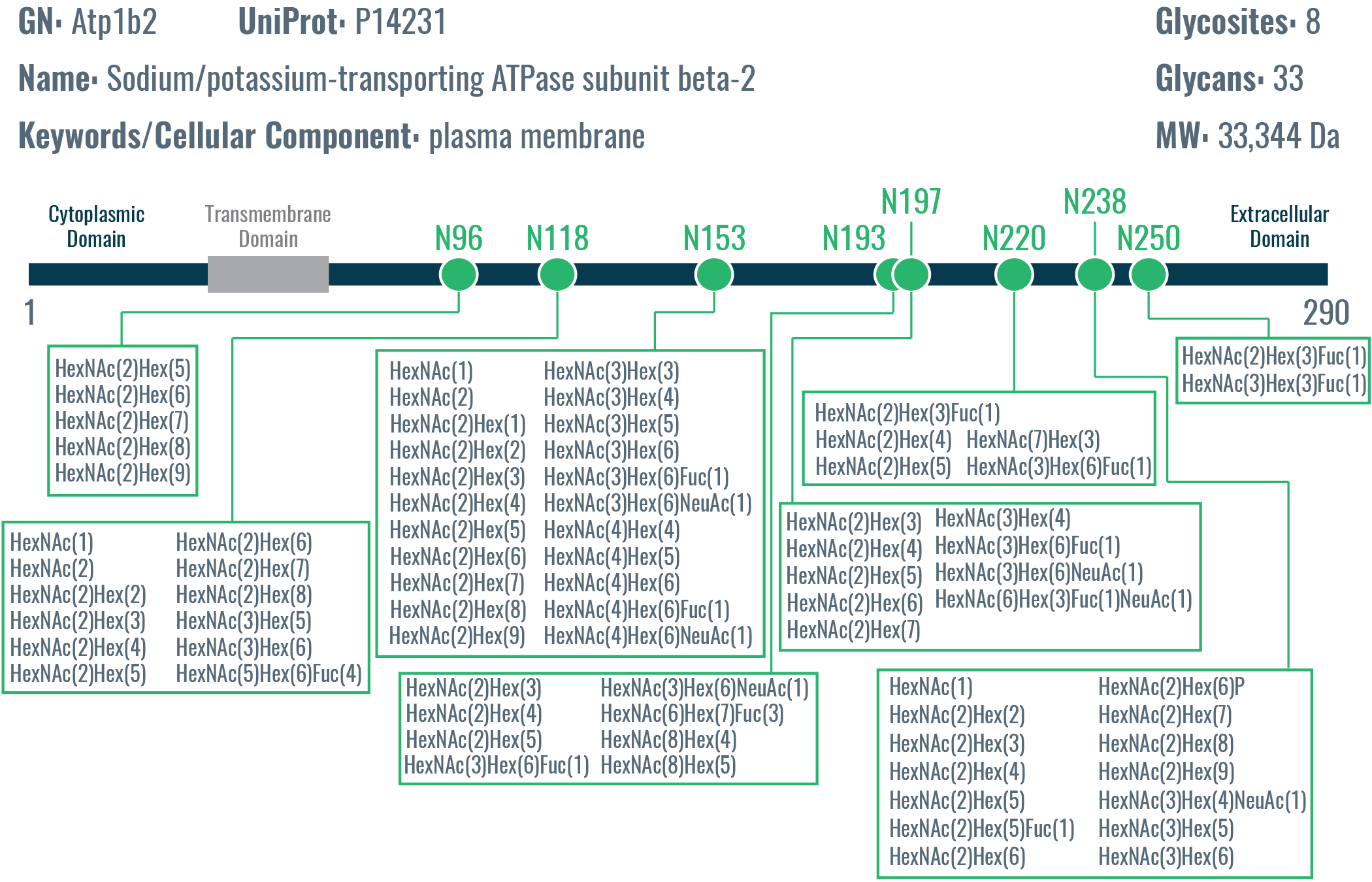


**Supplementary Figure 21. Sodium/potassium transporting-ATPase β2 subunit (Atp1b2): glycosites with varying degrees of glycan microheterogeneity.** Five of the eight glycosites display relatively low glycan microheterogeneity (<10 glycans), but N118, N153, and N238 show a higher degree of microheterogeneity 12, 22, and 14 localized glycans, respectively. Overall, this presents an interesting case were some glycosites display relatively little microheterogeneity while other glycosites on the same protein have a high degree of microheterogeneity.


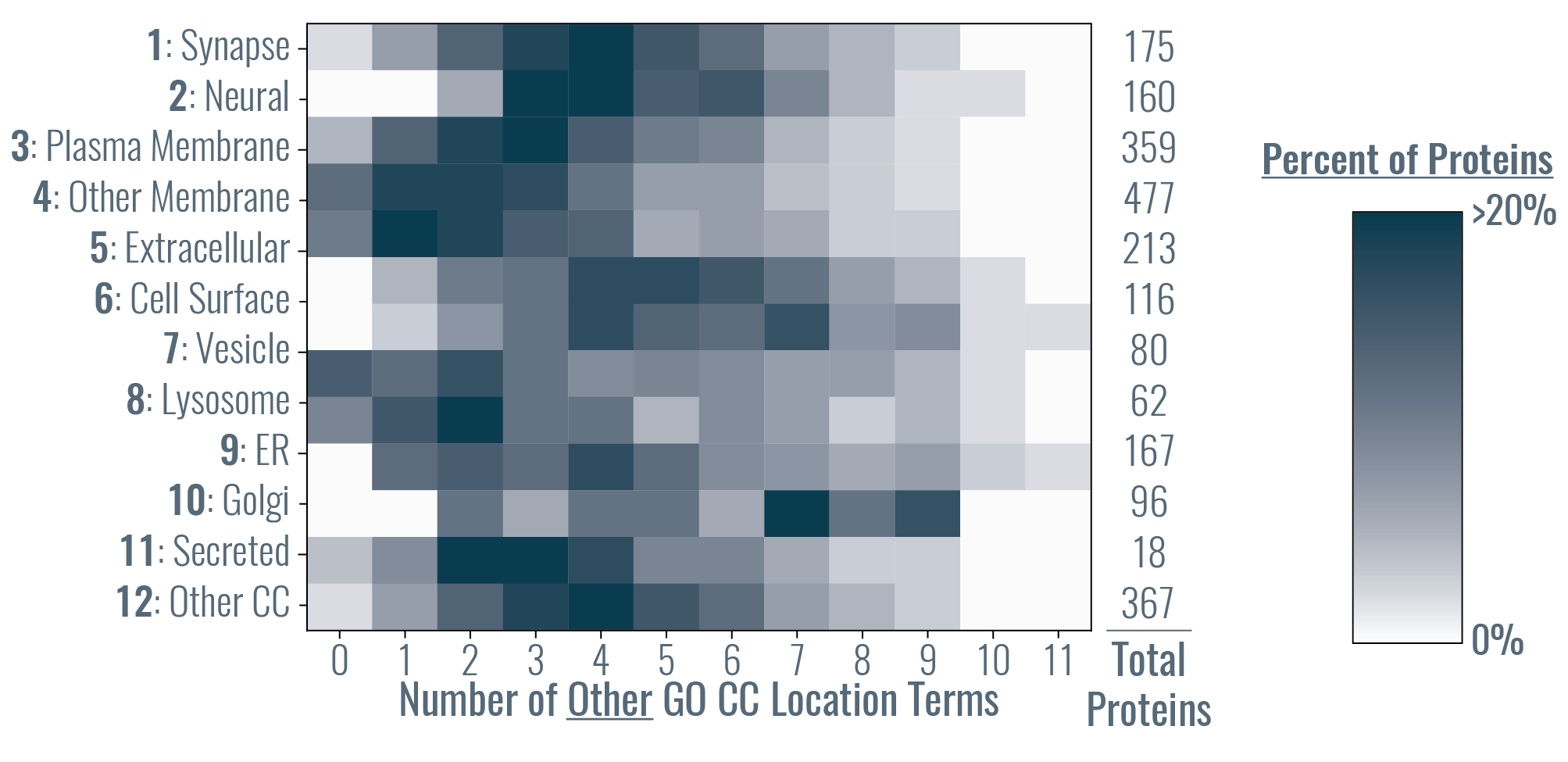


**Supplementary Figure 22. Heat map of the number of subcellular groups (derived from GO cellular component terms) for identified glycoproteins.** The y-axis shows the twelve subcellular groups (determined by GO cellular component terms associated with UniProt identification) and the x-axis is the number of GO “other” cellular component terms associated with a UniProt entry. For example, if a protein could be assigned to only one or two subcellular groups, the number of “other” location terms would be zero or one, respectively. The color of the heat map indicates the percentage of total protein identifications from that subcellular group that had a given number of other GO cellular component terms. This graph shows that a significant proportion of proteins could be classified as more than one subcellular group (i.e., location).

**SUPPLEMENTARY REFERENCES**

1. Halim, A. *et al.* Assignment of Saccharide Identities through Analysis of Oxonium Ion Fragmentation Profiles in LC–MS/MS of Glycopeptides. *J. Proteome Res.* **13,** 6024–6032 (2014).

2. Yu, J. *et al.* Distinctive MS/MS Fragmentation Pathways of Glycopeptide-Generated Oxonium Ions Provide Evidence of the Glycan Structure. *Chem. - A Eur. J.* **22,** 1114–1124 (2016).

3. Pett, C. *et al.* Effective Assignment of α2,3/α2,6-Sialic Acid Isomers by LC-MS/MS-Based Glycoproteomics. *Angew. Chemie Int. Ed.* **57,** 9320–9324 (2018).

4. Zielinska, D. F., Gnad, F., Wiśniewski, J. R. & Mann, M. Precision mapping of an in vivo N-glycoproteome reveals rigid topological and sequence constraints. *Cell* **141,** 897–907 (2010).

5. Fang, P. *et al.* In-depth mapping of the mouse brain N-glycoproteome reveals widespread N-glycosylation of diverse brain proteins. *Oncotarget* **7,** 38796–38809 (2016).

6. Liu, M. Q. *et al.* PGlyco 2.0 enables precision N-glycoproteomics with comprehensive quality control and one-step mass spectrometry for intact glycopeptide identification. *Nat. Commun.* **8,** 438 (2017).
