# Supplementary material for: Capturing site-specific heterogeneity with large-scale N-glycoproteome analysis
Source: Nat Commun. 2019 Mar 21;10:1311. doi: 10.1038/s41467-019-09222-w (PMC6428843; doi:10.1038/s41467-019-09222-w)
Supplement: Supplementary file 3 — Description of Additional Supplementary Files [file 41467_2019_9222_MOESM3_ESM.docx]

Description of Additional Supplementary Files

**Supplementary Data 1:** Glycopeptides.xlsx
Description: Information about identified glycopeptides from mouse brain tissue.

**Supplementary Data 2:** Glycoproteins.xlsx
Description: Information about identified glycoproteins from mouse brain tissue.

**Supplementary Data 3:** Glycosites.xlsx
Description: Information about identified glycosites from mouse brain tissue.

**Supplementary Data 4:** Glycans.xlsx
Description: Information about glycans seen on identified glycopeptides from mouse brain tissue.

**Supplementary Data 5:** GlycoPSMs.xlsx
Description: Information about all identified glycopeptide spectral matches from mouse brain tissue.

**Supplementary Data 6:** GlycoFocusedProteinDB.txt
Description: Fasta file that has proteins identified in the deglycoproteomics experiment. This database was used to search intact glycopeptide spectra.
