# Supplementary material for: Capturing site-specific heterogeneity with large-scale N-glycoproteome analysis
Source: Nat Commun. 2019 Mar 21;10:1311. doi: 10.1038/s41467-019-09222-w (PMC6428843; doi:10.1038/s41467-019-09222-w)
Supplement: Supplementary file 10 — Reporting Summary [file 41467_2019_9222_MOESM10_ESM.pdf]

nature research

Corresponding author(s): Joshua J. Coon

Reporting Summary

Nature Research wishes to improve the reproducibility of the work that we publish. This form provides structure for consistency and transparency in reporting. For further information on Nature Research policies, see Authors & Referees and the Editorial Policy Checklist.

Statistical parameters

When statistical analyses are reported, confirm that the following items are present in the relevant location (e.g. figure legend, table legend, main text, or Methods section).

n/a	Confirmed	
☐	☒	The exact sample size (n) for each experimental group/condition, given as a discrete number and unit of measurement
☐	☒	An indication of whether measurements were taken from distinct samples or whether the same sample was measured repeatedly
☐	☒	The statistical test(s) used AND whether they are one- or two-sided<br>*Only common tests should be described solely by name; describe more complex techniques in the Methods section.*
☐	☒	A description of all covariates tested
☐	☒	A description of any assumptions or corrections, such as tests of normality and adjustment for multiple comparisons
☐	☒	A full description of the statistics including central tendency (e.g. means) or other basic estimates (e.g. regression coefficient) AND variation (e.g. standard deviation) or associated estimates of uncertainty (e.g. confidence intervals)
☒	☐	For null hypothesis testing, the test statistic (e.g. F, t, r) with confidence intervals, effect sizes, degrees of freedom and P value noted<br>*Give P values as exact values whenever suitable.*
☒	☐	For Bayesian analysis, information on the choice of priors and Markov chain Monte Carlo settings
☒	☐	For hierarchical and complex designs, identification of the appropriate level for tests and full reporting of outcomes
☒	☐	Estimates of effect sizes (e.g. Cohen's d, Pearson's r), indicating how they were calculated
☒	☐	Clearly defined error bars<br>*State explicitly what error bars represent (e.g. SD, SE, CI)*

Our web collection on statistics for biologists may be useful.

Software and code

Policy information about availability of computer code

Data collection	Thermo Fisher Scientific Tune 3.0, Xcaliber 4.0, and Dionex Chromeleon 7.2 were used for data collection
Data analysis	Coon OMSSA Proteomic Analysis Software Suite (COMPASS) (https://www.ncbi.nlm.nih.gov/pubmed/21298793) and Byonic 2.11 were used for data analysis. C# scripts using C# Mass Spectrometry Library (CSMSL, available at https://github.com/-dbaileychess/CSMSL) were used for further data processing.

For manuscripts utilizing custom algorithms or software that are central to the research but not yet described in published literature, software must be made available to editors/reviewers upon request. We strongly encourage code deposition in a community repository (e.g. GitHub). See the Nature Research guidelines for submitting code & software for further information.

Data

Policy information about availability of data

All manuscripts must include a data availability statement. This statement should provide the following information, where applicable:
- Accession codes, unique identifiers, or web links for publicly available datasets
- A list of figures that have associated raw data
- A description of any restrictions on data availability

Raw data files (.RAW files) are available at online at the Chorus Project (chorusproject.org), Project ID: 1441. A free Chorus account is necessary to access this

April 2018

project: https://chorusproject.org/pages/dashboard.html#/projects/all/1441/experiments. Supplementary data files including identified glycopeptides, glycoproteins, glycosites, glycans, glycoPSMs, and proteins included in the glycoproteomics focused protein database – as well as Supplementary Information, Tables, and Figures – are available in the online version of the paper. Freely available Byonic results files containing all identified and assigned spectra are available at the following link: https://figshare.com/s/23abd7250324fbc81115. Note, these results files will contain identifications that were filtered out of the final dataset presented in this manuscript (using the post-Byonic filtering steps indicated above). Only spectral matches indicated in the provided glycoPSMs supplementary file were included in the dataset presented here. We recommend setting the "Max number of peaks per 100 m/z" to >50 (under "Annotation Options") to ensure annotation of all fragments in more complex spectra. Data have also been deposited to the ProteomeXchange Consortium via the PRIDE partner repository with the dataset identifier PXD011533.

Field-specific reporting

Please select the best fit for your research. If you are not sure, read the appropriate sections before making your selection.

☒ Life sciences ☐ Behavioural & social sciences ☐ Ecological, evolutionary & environmental sciences

For a reference copy of the document with all sections, see nature.com/authors/policies/ReportingSummary-flat.pdf

Life sciences study design

All studies must disclose on these points even when the disclosure is negative.

Sample size

Several replicate samples from one mouse brain

Data exclusions

No data were excluded, other than filtering the post-search Byonic data as described in the methods. These processing steps included: manual filtering to 1% false discovery rate (FDR) at the peptide spectral match level using the 2D-FDR score (Byonic typically retains identifications that are above the 1% FDR cutoff set in the Byonic software but pass protein FDR, especially for glycopeptides, which necessitates this step); removing identifications that had a Byonic Score below 150 (as suggested by Lee et al.); setting a threshold for peptide length at 5 residues or greater; and retaining glyco PSMs that had |logProb| value above 1 (which is the absolute value of the log base 10 of the protein p-value). This allowed for an estimated 0.33% FDR at the glycopeptide spectral match level (i.e., specifically counting the number of target and decoy hits that are glycopeptides, not including non-modified sequences). AI-ETD and HCD spectra had estimated FDRs of 0.07% and 0.59%, respectively. Furthermore, we removed glycopeptide identifications that contained more than one glycosite (because of known issues with properly assigning modifications in multiply glycosylated peptides). A further filtering step was added that only allowed for identifications with a Delta Mod Score of 10 or greater, which removed all decoy hits, and this pool of filtered identifications comprises the reported identifications in the manuscript.

Replication

Three technical replicates were collected for AI-ETD data, with one replicate each of ETD and EThcD for comparison (only one AI-ETD replicate was used to compare).

Randomization

Five technical replicates were collected total, with the three AI-ETD replicates being collected first followed by EThcD and then ETD data.

Blinding

Blinding was not relevant to our study because there were not treatment conditions for different samples, and fragmentation type needed to be known to properly process data.

Reporting for specific materials, systems and methods

Materials & experimental systems

n/a	Involved in the study
☒	☐ Unique biological materials
☒	☐ Antibodies
☒	☐ Eukaryotic cell lines
☒	☐ Palaeontology
☐	☒ Animals and other organisms
☒	☐ Human research participants

Methods

n/a	Involved in the study
☒	☐ ChIP-seq
☒	☐ Flow cytometry
☒	☐ MRI-based neuroimaging

Animals and other organisms

Policy information about studies involving animals; ARRIVE guidelines recommended for reporting animal research

Laboratory animals

C57BL/6, female, adult, 4 months old

Wild animals

The study did not involve wild animals.

Field-collected samples | The study did not involve samples collected from the field.
